# Basal ganglia–cortical interactions in Parkinsonian patients

**DOI:** 10.1016/j.neuroimage.2012.10.088

**Published:** 2013-02-01

**Authors:** André C. Marreiros, Hayriye Cagnan, Rosalyn J. Moran, Karl J. Friston, Peter Brown

**Affiliations:** a Nuffield Department of Clinical Neurology, University of Oxford, UK; b Sobell Department of Motor Neuroscience and Movement Disorders, Institute of Neurology, University College London, UK; c The Wellcome Trust Centre for Neuroimaging, University College London, UK

**Keywords:** BF, Bayes factor, DCM, dynamical causal modelling, DBS, deep brain stimulation, EEG, electroencephalography, GPe, Globus Pallidus externa, GPi, Globus Pallidus interna, IPSP, inhibitory postsynaptic potential, LFP, local field potential, MAP, *maximum a posteriori*, PCA, principal component analysis, PD, Parkinson's disease, STN, subthalamic nucleus, SVD, singular value decomposition, UPDRS, unified Parkinson's disease rating scale, VAR, vector auto regression, Basal ganglia, Parkinson's disease, Deep brain stimulation, Electroencephalography, Effective connectivity

## Abstract

Parkinson's disease is a common and debilitating condition, caused by aberrant activity in a complex basal ganglia–thalamocortical circuit. Therapeutic advances rely on characterising interactions in this circuit. However, recording electrophysiological responses over the entire circuit is impractical. Dynamic causal modelling offers large-scale models of predictive value based on a limited or partial sampling of complex networks. Using dynamic causal modelling, we determined the network changes underlying the pathological excess of beta oscillations that characterise the Parkinsonian state. We modelled data from five patients undergoing surgery for deep brain stimulation of more than one target. We found that connections to and from the subthalamic nucleus were strengthened and promoted beta synchrony, in the untreated compared to the treated Parkinsonian state. Dynamic causal modelling was able to replicate the effects of lesioning this nucleus and may provide a new means of directing the search for therapeutic targets.

## Highlights

► Novel model of basal ganglia-cortical system based on data from Parkinson’s patients. ► Model exposes criticality of connections to and from subthalamic nucleus in PD. ► Model able to replicate the effects of known surgical interventions in PD. ► Allows *in silico* tests of candidate therapeutic interventions in circuit disorders.

## Introduction

Parkinson's disease (PD) is associated with abnormally synchronised oscillations in the beta (~ 20 Hz) frequency band in the cortical–basal ganglia–thalamo–cortical loop (Hammond et al., 2007; Jenkinson and Brown, 2011; Uhlhaas and Singer, 2006). Treatment-induced reduction in the amplitude of these oscillations correlates with motor improvement (Jenkinson and Brown, 2011). Conversely, the artificial induction of beta oscillations slows movement in patients with PD (Chen et al., 2011; Eusebio et al., 2008) and healthy subjects (Pogosyan et al., 2009), and exacerbates Parkinsonian behaviour in rodents (Gradinaru et al., 2009). These observations suggest that high levels of beta activity could be mechanistically related to Parkinsonian motor impairments, or, at the very least, provide a faithful biomarker of the Parkinsonian state. Thus the network changes that underpin this activity may be highly informative about the pathophysiology of the disease and help direct the search for new treatment targets.

Yet, one of the major challenges in neurobiology is characterising dynamic interactions in complex and distributed networks – such as the cortical–basal ganglia–thalamo–cortical loop – that can only be partially sampled. Dynamic causal models (DCMs) allow electrophysiological data to be fitted by biologically plausible (conductance-based), neural-mass models of coupled sources (Moran et al., 2009). Importantly, complex models of neural circuits can be identified using data from just a subset of the components of these circuits (Moran et al., 2011). Crucially, inferences can still be made about the remaining circuit components, based on the influence they exert on the observed components. The importance of this novel modelling approach lies in the potential to identify new therapeutic targets and explore the effects of any interventions *in silico*. Here, we develop a DCM based on exceptional archival data from a group of PD patients who underwent simultaneous implantation of deep brain stimulation (DBS) electrodes into the Globus Pallidus interna (GPi) and the subthalamic nucleus (STN) and recording of electroencephalographic activity (EEG). Recordings were made both OFF and ON the dopaminergic prodrug, levodopa, to determine the key network differences between these states.

## Material and methods

### Patients

The patients gave informed consent to take part in the study, which was approved by the Ethical Committee of the CTO “A. Alesini” Hospital. All five patients (mean age of 50 years; range of 37–64 years; two females; mean duration of disease, 14 years; range of 9–24 years) were enrolled in a trial of combined pallidal and subthalamic DBS (Peppe et al., 2004). Their mean United Parkinson's Disease Rating Scale (UPDRS) motor scores were 66 (range of 48–80) and 13 (range of 7–20) off and on medication, respectively. Patients took a mean daily dosage of 950 mg of levodopa (range of 150–1500 mg). LFP features have been previously reported in all but one patient (Brown et al., 2001; Cassidy et al., 2002; Fogelson et al., 2005; Williams et al., 2002).

The operative procedure has been described previously (Brown et al., 2001; Peppe et al., 2004). Macroelectrodes were inserted after GPi and STN had been identified by non-telemetric ventriculography and localised using microelectrode recording and microstimulation whilst the subject was awake. The coordinates at the tip of contact 0 were 19–24 mm from the midline of the patient, 2 mm in front of the mid-commissural point, and 6 mm below the anterior commissure (AC)–posterior commissure (PC) line for GPi, and 12 mm from the midline, 0 mm from the mid-commissural point, and 4–5 mm below the AC–PC line for STN. Macroelectrode position was confirmed postoperatively using magnetic resonance imaging (MRI) or computerised tomography superimposed on pre‐operative MRI using image fusion systems. The DBS electrodes in the pallidum and STN were models 3387 and 3389 (Medtronic Neurological Division, Minneapolis, MN) with four platinum–iridium cylindrical surfaces. Contact 0 was the most caudal, and contact 3 was the most rostral.

### Electrophysiological recordings

Electrophysiological recordings were made 3–6 days postoperatively, in the interval between DBS electrode implantation and subsequent connection to a subcutaneous stimulator. Recordings were acquired whilst the patients were seated on a bed in a resting, awake state and both following overnight withdrawal of antiparkinsonian medication and about 1 h after 200 mg levodopa administration. Deep brain activity was recorded bipolarly from the adjacent four contacts of each DBS electrode (0–1, 1–2, and 2–3). EEG activity was recorded bipolarly from a single pair of EEG electrodes, either Cz–Fz or Cz–FCz (2 cases) using 9 mm silver/silver chloride electrodes or needle electrodes (3 cases). The ground was placed on a shoulder. Signals were amplified and pass band filtered between 1 and 300 Hz using a Nicolet Viking IIe and data captured through an A-D card (PCM-DAS12, ComputerBoards, Middleboro, MA 02346, USA) onto a portable computer using custom-written software. Signals were sampled at 1 kHz.

### Dynamic causal modelling for steady state responses

DCM provides a generic framework to infer the biophysical causes of neuroimaging data (Marreiros et al., 2010). Unlike functional connectivity measures such as correlations or coherence, which examine the statistical dependencies of time series data, DCM uses a generative or forward model to allow inferences about the underlying mechanisms behind the observations; that is, directed effective connectivity — a model based characterisation of causal influences. In the general case, DCMs describe how experimental manipulations (*u*) influence the dynamics of hidden (neuronal) states of the system (*x*), using the state evolution equation $\dot{x}=f\left(x\left(t\right),u\left(t\right),\theta \right)$, where *x* is the rate of change of the system's states *x*, *f* summarises the biophysical mechanisms underlying the temporal evolution of *x*, and *θ* is a set of unknown evolution parameters. DCMs map the system's hidden states (*x*) to experimental measures (*y*), typically written as the following static observation equation *y* = *g*(*x*,*φ*) where *g* is the instantaneous mapping from system states to observations and *φ* is a set of unknown observation parameters. Here, we use a DCM for steady state responses (SSR), which uses a generative model of a distributed network of interacting neuronal sources to predict observant spectral densities (Moran et al., 2009). The dynamics of these sources are specified by a set of first-order differential equations (Moran et al., 2011). DCM for SSR models the activity of a source with a neural mass model, which ascribes one or more subpopulations to each source (Supplemental Fig. S1). The activity of subpopulations are modelled with hidden neuronal states (ensemble depolarisation and firing rates), whose dynamics depend on intrinsic parameters that encode the amplitude of post synaptic responses and synaptic rate constants. The ensemble firing of one population drives the average membrane potential of others through either glutamate (which produces postsynaptic depolarisation) or GABA (hyperpolarisation) as a neurotransmitter. These effects are mediated by a postsynaptic (alpha) kernel that is either positive or negative. The (excitatory or inhibitory) influence of one subpopulation on another is parameterised by extrinsic effective connectivity (between sources) or intrinsic connectivity (within sources). Effective connectivity is modelled as a gain factor that couples discharge rates in one subpopulation to depolarisation in another. The model's architecture (see below) was identical to that used by us in our previous study in the 6-hydroxy-dopamine (6OHDA) midbrain lesioned rodent model of Parkinsonism (Moran et al., 2011). Three (layered) populations were used to model the cortical source (Moran et al., 2009), and a single population of neurons, either glutamatergic (excitatory) or GABAergic (inhibitory) was used for BG nuclei (Fig. 2). The model's prior values are provided in Supplemental Table 1. During Bayesian model inversion, these parameters are estimated in terms of posterior probability densities — summarised with their conditional mean and covariance (Supplemental Fig. S6). The posterior or conditional means of the connectivity and synaptic parameters are the most likely given the data (see Supplemental material for further information on DCM for SSR). In the present analysis, all the neural mass model parameters (Supplemental Table 1) were the same for the two conditions, ON and OFF levodopa, and the effect of levodopa was modelled by changes in the strength of extrinsic connections. Therefore, differences in observed spectral profiles were explained in terms of coupling changes amongst the nodes of the underlying network model, with a gain of more or less than one representing an increase or decrease, respectively, in connection strength.

### Model structure

The DCM was based on the motor cortico–basal ganglia–thalamo–cortical loop (Fig. 2). The connections were based on the well characterised re-entrant circuits linking the cortex, basal ganglia and thalamus, where the main features of this network include the so-called ‘direct’, ‘indirect’ and ‘hyperdirect’ pathways (Nambu, 2004; Smith et al., 1998). The cortex was modelled by a three layer cell ensemble which includes excitatory spiny stellate cells, projection (pyramidal) glutamatergic cells and inhibitory GABAergic interneurons. Excitatory projections from cortex innervate the striatum, and STN (the hyperdirect pathway). The striatum comprises an inhibitory cell mass that projects to other inhibitory cell masses, the GPe (indirect pathway) and GPi (direct pathway). The GPe is reciprocally connected to the excitatory cell mass of the STN (Bevan et al., 2002a,b). STN projects to GPi, which in turn projects (through another extrinsic connection) to the thalamus. The thalamus, which excites cortex, is itself inhibited by GPi. The major glutamatergic and GABAergic connections between six key components of the cortico–basal ganglia–thalamocortical circuit were thus incorporated into our standard model architecture (Smith et al., 1998). In particular, we included the two elements that have been promoted as crucial for the expression of exaggerated beta oscillations in Parkinsonism; the hyperdirect pathway (Gradinaru et al., 2009; Magill et al., 2001) and the reciprocal STN–GPe network (Cruz et al., 2011; Holgado et al., 2010). The effects of the experimental conditions, ON and OFF levodopa, were explained by the same model through changes in the extrinsic connections in the network. Although our standard model does not include all known connections, the addition of more connections does not necessarily improve the ability of the loop circuit to sustain beta oscillations (see comparison with other model architectures in the results).

### Recorded data and their analysis

The data used in this model were scalp EEG and LFPs from DBS electrodes in STN and GPi. The remaining areas were modelled as hidden sources. Note that by using DCMs, inferences can still be made about the parameters of hidden sources based on the influence they exert on nodes from which recordings are made. In fact, mathematically, all the parameters of a DCM are hidden or latent and the full dataset serves to optimise all of the parameters of the model. Brain activity recordings from cortex, STN and GPi were taken from the continuous time domain data. Principal component analysis (PCA) of multiple contacts in a single source or site was performed before spectral decomposition to reveal the internal structure of the data (*e.g.* from the three bipolar channels per site). PCA uses an orthogonal transformation (generally a *singular value decomposition* — SVD) to convert a set of observations of possibly correlated contacts into a set of values of uncorrelated variables which capture the greatest amount of variance expressed over time. These are called principal components. The first principal components, which account for as much of the variability in each data set as possible, were taken as the representative of the signals in GPi and STN. Frequency domain representations of LFP and EEG were constructed from the principle components and recorded time series, respectively using a vector auto regression (VAR) model of order p = 8. Specifically, channel data *y*, from the three channels (Cortex, STN and GPi) were modelled as an AR process.

$$y_{n}=A^{\left(1\right)}y_{n-1}+A^{\left(2\right)}y_{n-2}\ldots +A^{\left(p\right)}y_{n-p}+e$$

Note that principal component analysis produced a principle eigenvariate that was dominated by a single peak within the frequency band of interest. The model order, *p*, determines the number of peaks in the associated spectra, and our selection of *p* = 8 adequately accommodated these spectra, affording robust and smooth spectral features. In selecting a model order of 8 we reprised the model order used in a DCM analysis of LFP data from the lateral nucleus of the amygdala and the dorsal hippocampus (Moran et al., 2009), this order also gave the best results across the patient data. We also tried *p* = 14, but this tended to produce spectra in which the dominant peak was often divided in two. Model fits were slightly worse in this case. Frequency splitting (the appearance of a spurious spectral peak) is a recognised problem with AR methods when the model order is too high (Spyers-Ashby et al., 1998).

Both the autoregressive coefficients $A\left(p\right)\in R^{3\times 3}$ and channel noise covariance *E*_*ij*_ were estimated using the spectral toolbox in SPM (http://www.fil.ion.ucl.ac.uk) which allows for Bayesian point estimators. This entails a variational approach that estimates the approximated posterior densities in terms of conditional mean and covariance. These moments are optimised through hyperparameters encoding the precision of the innovations and the prior precision of the autoregressive parameters *per se* (Moran et al., 2009). The autoregressive coefficients and estimated channel noise covariance then provided a direct estimate of the cross-spectral densities, using the following transform:

$$\begin{array}{c} H_{\mathrm{ij}}\left(\omega \right)=\frac{1}{A_{\mathrm{ij}}^{\left(1\right)}e^{\mathrm{iw}}+A_{\mathrm{ij}}^{\left(2\right)}e^{i2w}+......+A_{\mathrm{ij}}^{\left(p\right)}e^{\mathrm{ipw}}}\\ g_{\mathrm{ij}}{\left(\omega \right)}_{c}=H{\left(\omega \right)}_{\mathrm{ij}}E_{\mathrm{ij}}H{\left(\omega \right)}_{\mathrm{ij}}^{*} \end{array}$$

We focused on the frequency window from 13 to 35 Hz as this is the frequency band that has been most clearly implicated in Parkinsonism in both correlative (Jenkinson and Brown, 2011) and causality (Chen et al., 2011; Eusebio et al., 2008; Gradinaru et al., 2009) studies.

## Results

### Spectral density model fits

**Fig. 1:**
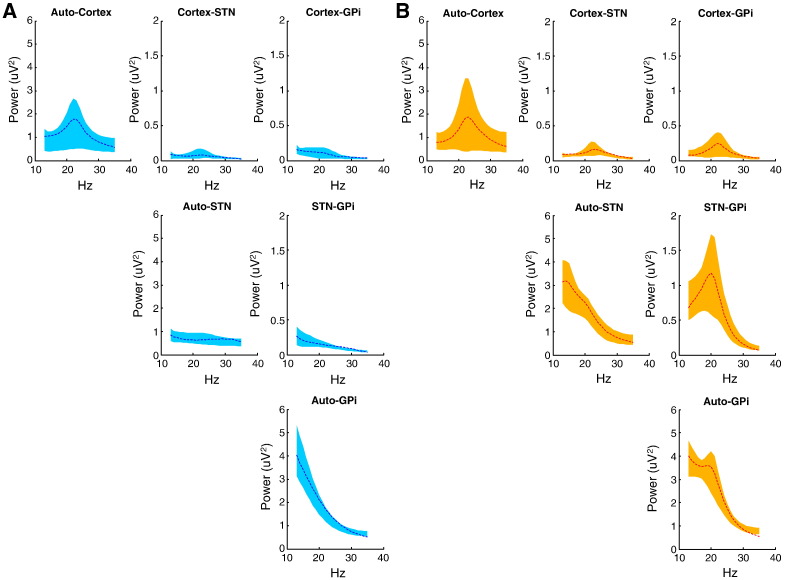
Model fit to recorded data. (A,B) Averaged auto-spectral and cross-spectral densities from cortex, subthalamic nucleus (STN) and Globus Pallidus internal (GPi) over 13–35 Hz (evaluated using a vector autoregressive model) ON (A) and OFF levodopa (B). Spectral responses averaged across the 12 data segment pairs from the five patients are plotted as dotted lines, whilst the 95% confidence intervals (CI) of the corresponding DCM predictions are shown as a shaded area. The main diagonal displays the auto-spectral densities at each site and the off-diagonal elements shows the cross spectra. All empirical spectra fall inside the shaded areas and indicate an overall good model fit.

We examined the frequency or spectral responses using resting state data from patients OFF and ON levodopa. Three segments, each of mean 50.6 ± (SEM) 2.4 s duration, were assessed for each drug state per patient. We used principal component analysis (PCA) to summarise the signals from the three bipolar contact pairs of each electrode in the STN and GPi and isolated the first component, which explained on average 74.4 ± 0.6% of the variance. A vector autoregressive model revealed a peak in power spectra over the beta 13–35 Hz band in both group-averaged EEG and in averages of the principal component of depth recordings from each site OFF levodopa across all patients (see Fig. 1A). These peaks were attenuated ON levodopa (Fig. 1B), as confirmed by a one-way ANOVA; with averaged beta activity pooled over auto and cross-spectra ON and OFF medication as a factor (F(1,23) = 14.15, p = 0.0011). Beta activity was pooled over auto and cross-spectra by way of data reduction prior to this ANOVA, but separate contrasts of ON and OFF for pooled auto-spectra (F(1,23) = 12.45, p = 0.0019) and pooled cross-spectra (F(1,23) = 11.43, p = 0.0027) afforded similar results. When auto-spectra were broken down still further to individual sites, only those for STN were significantly different between ON and OFF (F(1,23) = 34.28, p < 0.0001). Similarly, when cross-spectra were considered individually, only that for STN–GPi differed significantly between ON and OFF (F(1,23) = 15.21, p = 0.0008).

**Fig. 2:**
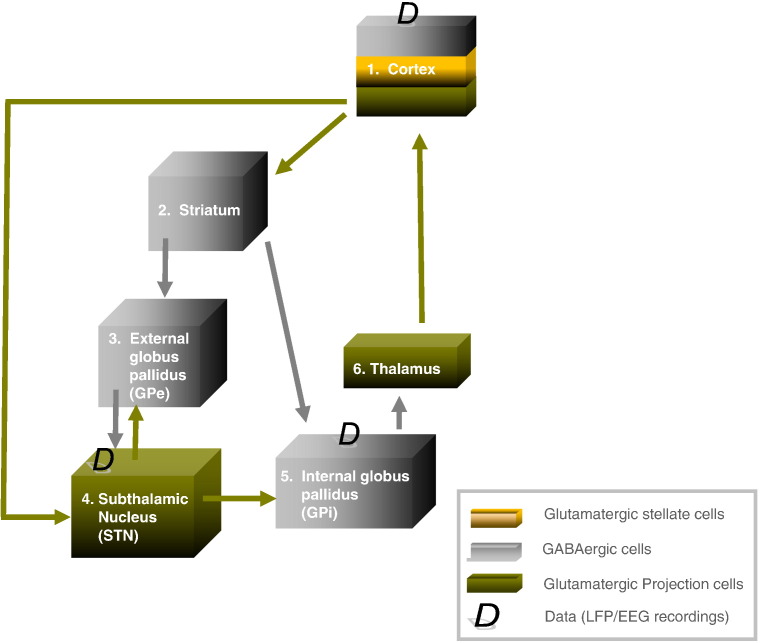
The dynamic causal model. The DCM comprised the principal nodes and connections in the human motor cortico-basal ganglia-thalamocortical loop: the nodes included motor cortex, modelled by a three layer cell ensemble comprising input, excitatory spiny stellate cells, projection (pyramidal) glutamatergic cells and inhibitory GABAergic interneurons. Excitatory projections from cortex innervated the Striatum, and STN (the hyperdirect pathway). The striatum comprised an inhibitory cell mass that projected to two other inhibitory cell masses, GPe (as part of the indirect pathway), and GPi (*via* the direct pathway). The GPe and STN expressed reciprocal connections, and signals from the hyperdirect and indirect pathways were conveyed via excitatory STN projections to the GPi. The thalamus, which excited the cortex, was itself inhibited by connections from GPi. Data, D, used for the model inversion were acquired from recordings in cortex, STN and GPi. For model parameter's prior values see Supplemental Table 1.

ON and OFF medication data segments were then paired and their cross-spectral densities were modelled using the DCM illustrated in Fig. 2. This was repeated for each of three segment pairs per patient. Model fitting or inversion entails estimating the mean and variance of unknown model parameters using the spectral density data-features (see Supplemental material for further information on Bayesian model inversion and comparison). These unknown parameters include the biophysical parameters of the neural-mass model as well as parameters controlling the spectral composition of neuronal and channel noise. DCM fitted each pair of segments together, and only the strengths of the extrinsic (between-source) connections were allowed to change to account for the influence of levodopa. Supplemental Fig. 2 shows the model evidence across the three paired segments per patient of ON and OFF data. The overall fit (accuracy) of the DCM was consistent for all but three segment pairs, which were precluded from subsequent analysis (Supplemental Fig. 2). This left 12 pairs (with at least one from each subject). Model data were averaged from these 12 segment pairs to give the group mean model data (Fig. 1). Original data and model auto and cross spectra corresponded well for both ON (Fig. 1A) and OFF (Fig. 1B) levodopa states.

### Changes in effective connectivity between states

**Fig. 3:**
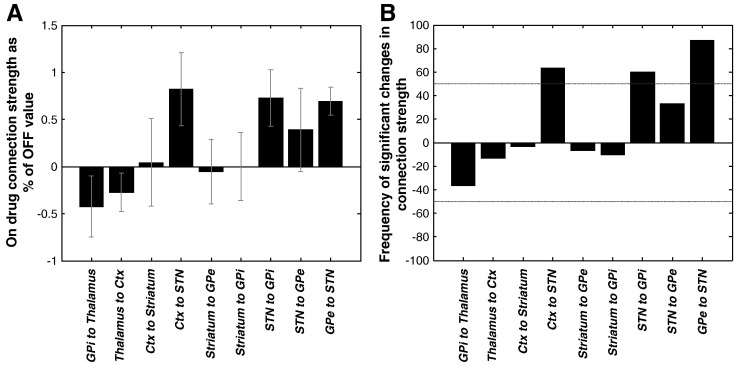
Changes in connectivity estimates between ON and OFF states. (A) The conditional probability that the ratio of *maximum a posteriori* (MAP) connection strengths (OFF/ON) is greater or smaller than one (pooled over the group sample) are shown with 95% CI. (B) Percentage of data segments (*n* = 12 segment pairs from the five patients) in which changes in connectivity between ON and OFF exceeded 95% confidence limits. The horizontal lines denote ≥ 50% of data segment pairs showed significant increases or decreases in connection strengths between states. Ctx is cortex; otherwise the abbreviations are as in Fig. 2.

The differences between ON and OFF levodopa states were modelled through the modulation of all extrinsic connections in the network. The connectivity *maximum a posteriori* (MAP) estimates for the 12 segments were combined and averaged over the group. Group differences between MAP (connectivity strength) estimates ON and OFF levodopa are illustrated in Fig. 3A for the nine extrinsic network connections. These were obtained through a simple average of the conditional means and their confidence intervals. Effective connectivity could increase or decrease; therefore, we defined significant changes as those in which [i] there was a 95% confidence about changes at the group level (with > 95% of the posterior mass OFF being above or below the level ON) and [ii] these changes were significant in at least 50% of the 12 individual segment pairs (Fig. 3B). The latter criterion was important as there was some variability in the spectral profiles and ensuing MAP estimates amongst the segment pairs. Some of this variability arose at the subject level and might relate to slight differences in surgical targeting, surgical stun effects or clinical phenotype. The variability between samples from the same subject might relate to slightly different levels of arousal at rest (see Suppplementary Results; Model validity and reproducibility). Under these criteria, the connections from GPe (Globus Pallidus externa) to STN (part of the indirect pathway), cortex to STN (hyperdirect pathway) and STN to GPi increased from ON to OFF levodopa. In order to preclude identifiability issues, we examined the posterior correlations amongst parameters from all DCMs. When high dependencies exist between two parameters, a change in either could account for the same differences in the data features. However, only small conditional correlations (0.06 ± 0.01) were observed amongst changes in extrinsic connectivity (Supplemental Fig. S5). Our model also produced well-behaved posterior densities, when compared to the equivalent prior densities (Supplemental Fig. S6).

### Comparison with other model architectures

**Fig. 4:**
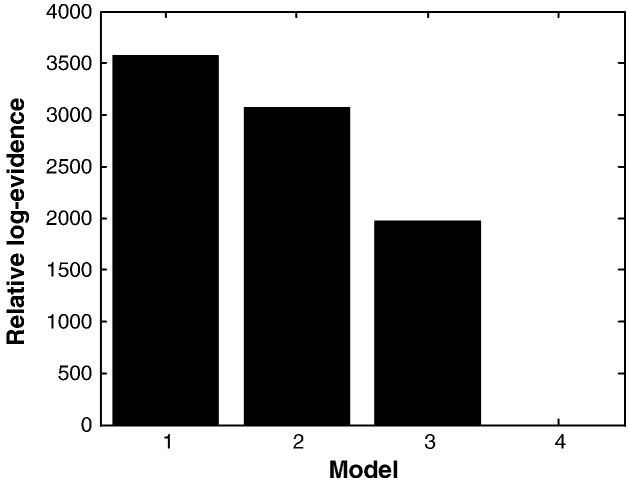
Bayesian model comparison. Fixed effect model comparison using data from all five patients, ON and OFF levodopa. Model 1 comprised the “standard” basal–ganglia–thalamocortical re-entrant circuit shown in Fig. 2. Model 2 included a new connection from GPe to GPi, whilst model 3 included a new connection from GPe to striatum. Model 4 comprised the “standard” architecture but with the data recorded from the electrode presumed to be in GPi now assigned to a source in GPe. All models are compared to Model 4. There is very strong evidence in favour of Model 1 — the standard model.

Our model architecture did not include all known connections. However, the addition of more connections might not necessarily improve the ability of the basal ganglia cortical circuit (and model) to generate beta oscillations. To address this issue, we tried adding two less well-studied, but potentially important, pallidofugal connections, either from GPe to GPi or from GPe to striatum (Bevan et al., 1998) and evaluated the evidence for these extra connections using Bayesian model comparison. Bayesian model comparison uses the (variational free energy approximation to) model evidence to compare competing hypotheses about the neural architecture generating data. For each of the 12 paired segments, we inverted or fitted the data using models with extra connections. In line with our previous findings in the Parkinsonian rodent (Moran et al., 2011), the addition of either connection did not increase model evidence, in relation to the standard model (Stephan et al., 2009). In addition, we tested a further model which comprised the “standard” architecture but in which we assumed that the signal ascribed to the GPi was actually derived from a source in GPe. This model was considered because (even with an optimally placed DBS electrode) the upper bipolar contact pair could sample the GPe rather than the GPi. This model performed poorly, consistent with our initial supposition that the pallidal signals arose from a GPi source. Fig. 4 confirms the strong evidence in favour of our original model (the standard model — Model 1). The Bayes Factor comparing the first two models was (*BF*_1,2_) >> 150. This corresponds to a >> 99% probability that the standard model was the most likely given our data (see Supplemental material for further information on Bayesian model inversion and comparison). We conclude that the model architecture described in Fig. 2 provided the best balance of accuracy and complexity for our given data set. Having established the adequacy of the basic model, we next quantified the contribution of different connections to the generation of beta activity:

### Contribution analysis

**Fig. 5:**
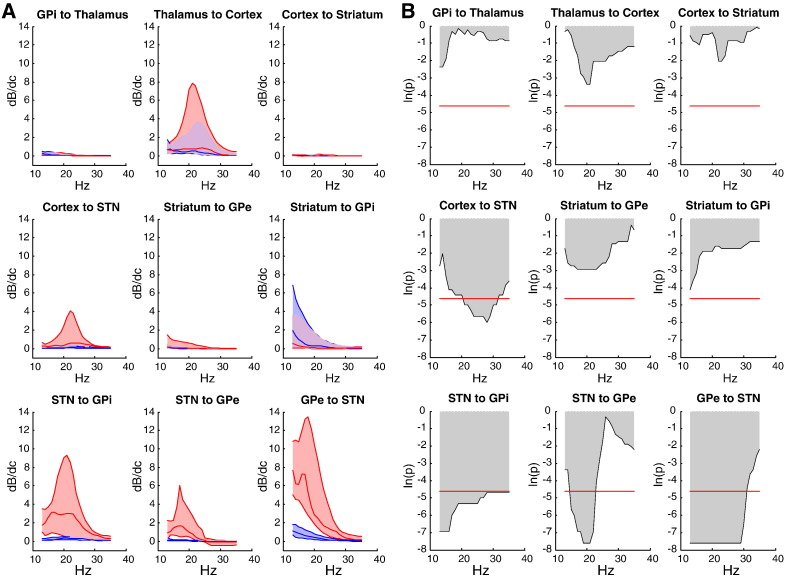
Contribution analysis. (A) Median contribution analysis for the spectral responses (across the beta frequency window), with respect to changes in connectivity parameters ON (blue) and OFF (red) levodopa. Respective lower and upper quartiles of the contribution results from the 12 DCM are shown as shaded areas. Positive changes mean that small increases in the respective connectivity strength produced higher beta activity in the Parkinsonian network. (B) Wilcoxon signed-rank test of the contribution analysis for ON, relative to OFF levodopa. Beta promoting potency was greater OFF than ON for four connections: cortex to STN, STN to GPi, STN to GPe and GPe to STN (p < 0.01 as shown by red horizontal lines in (B)).

We used the MAP estimates from our optimised DCMs above to investigate which connections promoted or attenuated beta-activity in the patients. Our goal was to see if particular connections contributed more to beta activity than others. We therefore quantified the degree to which a change in a coupling parameter, *c*, affected beta band oscillatory activity, *β*, throughout the circuit (Moran et al., 2011). The derivative, *dβ/dc*, was computed for beta responses (over a range of frequencies) at each source and averaged to create a measure of distributed beta-contribution for each connection. Fig. 5 shows the results of this contribution analysis (Fig. 5A) and statistical significance (Fig. 5B) of the differences in contribution ON and OFF levodopa, over the 12 segment pairs. Wilcoxon signed-rank tests across the contribution spectra revealed significantly (p < 0.01) different effects of contribution between the ON and OFF levodopa states for four key connections: cortex to STN, STN to GPi, STN to GPe and GPe to STN. For all of these connections, there were broad bands of beta range frequencies over which beta activity was promoted in the OFF compared to the ON levodopa state. Interestingly, these connections included the three with the greatest increases in effective connectivity in the OFF levodopa state (Fig. 3A) and were all linked to the STN. This suggests that increases in the connections linking STN with other regions are particularly important in exacerbating beta oscillations in our patients.

### Lesion analysis

**Fig. 6:**
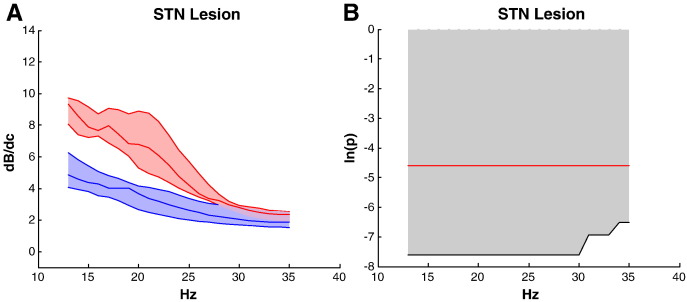
Lesion analysis. (A) Effect of lesioning all connections to and from STN on net beta activity in the Parkinsonian network (red — without lesion: blue — with lesion). The shaded areas correspond to lower and upper quartiles of the contribution results from the 12 DCM. (B) Wilcoxon signed-rank test of the effects on lesioning: beta activity was profoundly and significantly suppressed. Horizontal red line denotes p < 0.01. Results were identical if the population of STN neurons was removed from the model.

STN lesions ameliorate Parkinsonism and suppress beta activity (Chen et al., 2006). Thus to test the face validity of our model, we simulated a STN lesion by setting the strengths of the connections to and from the STN to zero, and also by removing the STN source from the model altogether. The effects were identical, namely a profound attenuation of beta activity exhibited by the circuit (Fig. 6). We also explored the effects of separately setting each connection strength to and from STN to their respective ON levodopa values, whilst leaving all other connections with their OFF drug strengths (Supplemental Fig. S7). Even such partial lesioning of the GPe–STN and STN–GPe connections was sufficient to profoundly attenuate beta activity. Although partial lesioning of the CTX–STN and STN–GPi connections also attenuated circuit beta activity, these changes did not reach significance at the group (sample) level. Complete lesioning of these connections did, however, achieve significant attenuation of beta activity in the system (Supplemental Fig. S7).

## Discussion

Classical models of connectivity within the cortico–basal ganglia–thalamocortical circuit explain PD symptoms in terms of altered firing rates along the direct/indirect pathways (DeLong, 1990). More recent research has highlighted the role of pathological oscillatory synchronisation in the Parkinsonian state, particularly that in the beta frequency band (Hammond et al., 2007; Jenkinson and Brown, 2011; Uhlhaas and Singer, 2006). Our understanding builds on this through dynamic causal modelling of LFPs and EEG, which, in order to be detected, necessitate spatiotemporal summation and hence, synchronisation of activity, across local neuronal elements. Using neural mass models that embody ensemble firing output and membrane potential inputs, our model generated spectral activity patterns (that characterise PD patients OFF and ON medication) in terms of changes in the effective connectivity between particular nodes in the cortico–basal ganglia–thalamocortical circuit. Specifically, patients OFF medication had increased input to the STN via the hyperdirect pathway and from the GPe, as well as a strengthening of projections from the STN to the GPi. These connections, together with those from STN to GPe, also promoted beta oscillations within the network reconfigured by diminished tonic dopaminergic activity.

### Model architecture

Our model architecture, like others, represents an informed reduction of complex biological connectivity, and as such might not be the only architecture that can sustain exaggerated beta oscillations. However, our model does incorporate the major glutamatergic and GABAergic connections between the six key components of the circuit, thus capturing the core elements of the direct, indirect and hyperdirect pathways and placing it within established frameworks. Moreover, our standard model was sufficient to explain the pattern of beta activity recorded in the ON and OFF drug states and performed better than two more complex models in fitting two very different data sets; one from the Parkinsonian patients reported here and one from anaesthetised Parkinsonian rodents (Moran et al., 2011). Crucially, our model was also successful in making valid predictions regarding the consequence of lesions of the subthalamic nucleus and its connections. Lesioning, micro-lesioning or muscimol inactivation of this nucleus reduces beta oscillations and improves Parkinsonism in primates, including humans (Chen et al., 2006; Tachibana et al., 2011).

### Changes in connection strength between ON and OFF drug states

We found significant and relatively consistent differences in effective connectivity between the treated and untreated Parkinsonian states. First, the effective connection strength of the cortical ‘hyperdirect’ input to the STN was increased in the OFF state. Such a strengthening of the hyperdirect pathway in the OFF drug state would be consistent with recent fMRI findings in patients with PD (Baudrexel et al., 2011). It would also be in accord with experimental studies in the Parkinsonian rodent (Dejean et al., 2008; Magill et al., 2001) and reprises the critical role of the glutamatergic hyperdirect pathway in other models of PD (Holgado et al., 2010; Leblois et al., 2006). In addition, our previous DCM study (Moran et al., 2011) identified the hyperdirect pathway as strengthened in the Parkinsonian rodent, but as we contrasted healthy rodents with Parkinsonian animals treated with 6OHDA, we were unable to ask whether this represented an acutely reversible change in the hyperdirect pathway or a fixed consequence of chronic plasticity. The present findings confirm that the strengthening of the hyperdirect pathway in the Parkinsonian state is reversible by treatment with the dopamine prodrug, levodopa. This too finds a precedent; synaptic release of glutamate (and GABA) are suppressed by the activation of presynaptic D2 dopamine receptors in the STN (Baufreton and Bevan, 2008; Cragg et al., 2004; Shen and Johnson, 2000) and in experimental PD AMPA (and GABA) receptor agonists generate larger currents in postsynaptic STN neurons (Shen and Johnson, 2005).

Second, the GPe–STN connection was strengthened in the OFF state, consistent with the key role of over-activity in the indirect pathway in PD (Bergman et al., 1990; Kravitz et al., 2010). The loss of dopamine in the STN in PD may amplify GABAergic feedback inhibition from the GP (Cragg et al., 2004; Shen and Johnson, 2000, 2005). IPSPs due to GPe input are necessary to relieve the inactivation of Na_v_ channels during autonomous activity in the STN (Baufreton and Bevan, 2008), and may even promote rebound bursts by bringing the membrane potential to a more hyperpolarised state, which primes low-threshold calcium channels (Bevan et al., 2000). The interaction of increased GPe inhibition with autonomous pacemaker activity in STN is able to generate spontaneous oscillations at sub-beta frequencies *in vitro* (Baufreton et al., 2005; Bevan et al., 2002a,b; Plenz and Kital, 1999). *In vivo*, it has been proposed that the same increased GPe inhibition acts to potentiate cortically driven higher frequency oscillations in the STN (Baufreton et al., 2005), or, in the setting of tonic excitation of the STN by the cortex, enables the STN–GPe circuit to oscillate at higher frequencies (Holgado et al., 2010; Kumar et al., 2011). Hence the blockade of glutamatergic inputs from cerebral cortex (and thalamus) in the STN suppresses beta activity in the 1-methyl-4-phenyl-1,2,3,6-tetrahydropyridine (MPTP) treated primate (Tachibana et al., 2011).

### Effects of manipulating connection strengths

However, simple contrasts of the steady-state networks describing patients OFF and ON levodopa need not necessarily capture the functional significance of all connections in the OFF state. This is because connections might have profound influence upon abnormal activity in the re-organised ‘OFF’ circuit, even though their connection strengths might remain relatively unchanged between ON and OFF states. It is also important to clarify whether increases in effective connectivity are pathological and promote beta synchronisation or are secondary compensatory phenomena acting to counterbalance excessive beta synchrony. Contribution analysis can identify those connections whose beta promoting potency is much greater when embedded in the ‘OFF’ network. In our work, the tendency to promote beta activity was quantified by the derivative dβ/dc in response to small changes in connection strength. Small changes have the advantage that they are more likely to be assimilated within the circuit without reconfiguration to a new steady-state. This approach revealed that all three connections that are increased in the OFF state are also promoting beta synchrony, as did the connection from STN to GPe in the OFF state circuit. This suggests that the strengthening of the three connections is primarily pathological and not compensatory. In addition, the reciprocal connections between GPe to STN emerge as having a particularly important role in promoting beta synchrony in the ‘OFF’ compared to the ‘ON’ state circuit. The critical role of the GPe-STN circuit in maintaining beta is supported by previous modelling (Holgado et al., 2010; Kumar et al., 2011), recent experiments in 6OHDA midbrain lesioned rodents (Mallet et al., 2008a,b) and MPTP treated primates (Tachibana et al., 2011). In the latter instance, separate inactivation of the GPe and STN with muscimol was sufficient to suppress beta oscillations. The increased beta promoting effects of the STN-GPe and STN to GPi connections in the OFF state may relate to dopamine's D2/3-like presynaptic and D4-like post-synaptic receptors in the pallidum, which act to reduce excitatory input (Hernández et al., 2006), in combination with the functional effects on inhibitory input in the STN noted earlier.

With a rich recurrent architecture there may be several potential mechanisms for generating beta oscillations. One striking aspect of our results is the relatively modest role of connections to and from the striatum, especially given the florid dopaminergic denervation of the striatum in PD. Some modelling studies have emphasised the roles of the striatum and direct and indirect pathways (Gittis et al., 2011; Kumar et al., 2011; Leblois et al., 2006; McCarthy et al., 2011), although only those implicating the striatum and indirect pathway explicitly focussed on beta synchrony. The results of experimental studies have been rather variable. For example, a recent investigation in MPTP treated primates found that microinjection of gabazine into the GPe to block GABAergic inputs from the striatum failed to change beta in the GPe (Tachibana et al., 2011). In contrast, direct infusion of the cholinergic agonist carbachol into the striatum of otherwise healthy mice is able to induce prominent beta frequency oscillations in the striatal LFP (McCarthy et al., 2011). But rather than contest the importance of different elements in promoting beta oscillations, it seems reasonable to acknowledge that many interventions may have the potential to modulate beta synchrony; the issue is whether they reflect those changes that support beta oscillations in the disease state. In this regard, the results reported here are likely to be particularly relevant as our DCM was constrained to fit the pattern of synchronisation across nodes expressed in a comprehensive set of simultaneously acquired signals in patients with PD. This is not to say that even those elements that do not, in practice, contribute to beta synchrony are irrelevant, for they may well be important in modulating discharge rate, bursting and synchronisation at other frequencies.

Finally, we tested the face validity of our model by mimicking the effects of a STN lesion, and confirmed our expectation that this would attenuate beta activity across the basal ganglia–thalamocortical system (Chen et al., 2006). In addition, we explored the importance of each connection to and from the STN. Simply resetting the strength of either the GPe–STN or STN–GPe connections to their respective ON levodopa values, whilst leaving all other connections with their OFF drug strengths was sufficient to profoundly attenuate beta activity, underscoring the importance of the STN–GPe recurrent circuit in sustaining beta oscillations. The hyperdirect CTX–STN and the STN–GPi connections were also important in sustaining beta oscillations, although these connections had to be lesioned in order to achieve significant attenuation of beta activity in the system (Supplemental Fig. S7). The implication here is that although the STN–GPe recurrent circuit may be an essential resonator in the Parkinsonian system, it is not by itself sufficient to drive the exaggerated beta state; cortical activity is necessary as a substrate to be amplified or as a tonic input to maintain the STN–GPe circuit in a resonating mode (Holgado et al., 2010). Likewise, the dependency on the hyperdirect pathway rules out the STN *per se* as the beta generator. Indeed, even if STN–GPe was modelled using three interconnected subpopulations similar to the cortex (to explicitly provide a subcortical structure potentially capable of generating oscillations), it was still dependant on cortical input and in the absence of this input it was not able to generate the beta oscillations.

### Comparison with DCM in the 6OHDA midbrain lesioned rodent

Some of the major findings in the patients accord with those of our previous DCM study in Parkinsonian rodents (Moran et al., 2011). Both demonstrated strengthening of the hyperdirect pathway in the Parkinsonian state and increased beta promoting potency in the GPe to STN pathway. Differences did however exist (Supplemental Table 2). Indeed, it would have been surprising if there had not been some differences between the patient and rodent models, as they are based on data from different species and impairments underscored by progressive degeneration and acute toxicity, respectively. In particular, the treated Parkinsonian patient and healthy rodent cannot be considered strictly homologous. There are a number of important plastic changes that occur secondary to chronic dopaminergic cell loss that might have been apparent in a comparison between healthy and Parkinsonian animals, but not in one between Parkinsonian patients differing only in their treatment state (Surmeier et al., 2010). Conversely, plastic changes may also occur due to chronic intermittent dopaminergic therapy in patients that would be absent in the untreated Parkinsonian rodent (Chase, 2004). Neither is the profile of neurotransmitter loss and cell damage in PD as simple as in the 6OHDA midbrain lesioned rodent (Halliday and McCann, 2009). In addition, the effect of recent surgery should not be forgotten in the patient group. This is acknowledged to lead to microlesional or stun effects that may temporarily attenuate both beta synchrony and motor deficit (Chen et al., 2006). Finally, it should be stressed that the data used in our original rodent model were recorded in anaesthetised animals, whereas patients were alert during the recording.

### Comparison with other basal ganglia models

Several computational studies have investigated the oscillatory nature of activity in the basal ganglia network utilising forward models (Gillies et al., 2002; Holgado et al., 2010; Humphries et al., 2006; Leblois et al., 2006; Terman et al., 2002). Holgado et al. (2010), Gillies et al. (2002) and Terman et al. (2002) modelled the STN–GPe circuit and investigated changes that would occur in the network as synaptic weights were varied to capture the difference between the physiological and Parkinsonian state. In their seminal study, Holgado et al. reported that the STN–GPe circuit is capable of generating beta oscillations when three conditions are met: (1) STN–GPe and GPe–STN connections are strong enough, (2) excitation through the hyperdirect pathway is stronger than striatal inhibition on GPe and (3) the time required by neurons to react to their inputs needs to be short relative to synaptic transmission delays. Our findings were broadly similar, despite the fact that we considered a more extensive architecture (containing the direct, hyper-direct and indirect pathways) and our contrasts involved the change from the OFF drug to the ON drug state. Additionally, we highlight the hyperdirect pathway as a core element of the beta generating circuit, the former being given fixed connection strengths in the study of Holgado et al. (2010). Gillies et al. (2002) and Terman et al. (2002) also studied the STN–GPe network using firing rate and a conductance based models, respectively. Gillies et al. found recurrent connectivity within STN plays an important role in oscillation generation. Such connectivity was not included by Holgado et al., nor in the present study, as its existence is unclear (Hammond and Yelnik, 1983; Sato et al., 2000), and its incorporation does not seem essential for the generation of oscillations in the beta frequency band. Terman et al. (2002) reported that the STN–GPe network was capable of generating oscillations in the theta range, but their model did not include transmission delays between different nuclei, nor the hyper-direct input from cortex to STN.

Humphries et al. (2006) used a spiking neuron model to study oscillatory activity in the cortico–basal ganglia network in the dopamine depleted but *anaesthetised* state, which does not show beta oscillations (Magill et al., 2001; Mallet et al., 2008b), and in the intact, alert animal, where they found gamma activity. Leblois et al. (2006) included the hyper-direct and direct circuits of the cortico–basal ganglia network in their systems-level model and observed oscillations in the theta and alpha frequency bands. This model might not have been able to generate oscillations in the beta band due to the absence of the indirect pathway, which seems to be essential for the generation of oscillations at these frequencies.

Thus a number of methodological differences exist between studies, but the fundamental difference between the current and other modeling studies is the subject: we set out to contrast the pathophysiology of ON and OFF medication states by fitting our model to experimental data derived from patients rather than compare healthy and chronically dopamine depleted states based on parameters drawn from studies in non-humans (Gillies et al., 2002; Holgado et al., 2010; Humphries et al., 2006; Kumar et al., 2011; Leblois et al., 2006; McCarthy et al., 2011; Terman et al., 2002). Our goal was to derive a model that was of clinical value and would allow exploration of the effects of candidate therapeutic interventions through simulation, as demonstrated by our mimicking of the effects of a STN lesion. We reasoned that a model convolved with electrophysiological data from patients would have the best chance of capturing the effects of the complex profile of neurotransmitter loss and cell damage in PD and of any plastic changes due to chronic intermittent dopaminergic therapy, in so far as these will be expressed in the steady-state dynamics of the basal ganglia — cortical circuit. Such factors are not captured in models that contrast the physiological state with experimental Parkinsonism in non-humans. Nevertheless, despite these and the methodological differences discussed above, many of these models, as ours, highlight the central role of the STN–GPe circuit in the elaboration of pathological oscillations (Gillies et al., 2002; Holgado et al., 2010; Humphries et al., 2006; Kumar et al., 2011; Moran et al., 2011; Terman et al., 2002).

### Limitations and future developments

We must emphasise several limitations of our approach. First, the model presented here only provides a description of network dynamics that might subtend beta oscillations in the basal ganglia–cortical loop, predicated on the idea that these oscillations are important in elaborating the bradykinetic-rigid phenotype (Hammond et al., 2007; Jenkinson and Brown, 2011). Accordingly, we cannot comment on whether changes in connections might be important in sustaining other non-oscillatory or oscillatory activities, such as tremor.

Second, we should stress that connectivity changes were not entirely consistent between patients. Those considered here were significant in at least 50% of cases. Thus we may have overlooked meaningful variation in circuit characteristics. In the future, it will be important to define any relationship between less consistent changes in connectivity and either slight variations in surgical targeting or clinical phenotype in a study with larger numbers of patients.

Third, projections from the thalamus to the STN and globus pallidus and those between these latter sites and the pedunculopontine nucleus are likely to be important and were omitted from our model architecture. This should be addressed in future iterations using Bayesian model comparison, if and when simultaneous recordings of activities at these sites become available in patients.

Fourth, we did not allow neural mass model (synaptic) parameters to change between the ON and OFF levodopa conditions. In other words, we constrained the effect of levodopa to be exerted through changes in extrinsic coupling. This provided a parsimonious (efficient) model of the changes that was sufficient to answer our questions. In the future it would be interesting to consider an extended model space and test for differences in the intrinsic properties of the neuronal populations (although our initial analyses along these lines were confounded by convergence problems that sometimes attend over parameterised models).

### In perspective

Our analyses lead to a new view of connectivity in the basal ganglia–thalamocortical circuit, which acknowledges the importance of synchrony in the pathophysiology of Parkinson's disease. Our scheme makes strong and testable inferences about which projections have altered strategic importance in the pathological state and offer themselves as candidate therapeutic targets. Key amongst these strategically important connections are those to and from the STN. Of note, however, was that although the STN-GPe recurrent circuit may be an essential resonator in the Parkinsonian system, it still requires some degree of input through the hyperdirect pathway to operate in this mode. Finally, the approach developed here can be extended to other complex neural circuits, thereby allowing the exploration of the effects of candidate therapeutic interventions through tractable, safe, cheap, but valid simulations.
