# Supplementary material for: Basal ganglia–cortical interactions in Parkinsonian patients
Source: Neuroimage. 2013 Feb 1;66:301–10. doi: 10.1016/j.neuroimage.2012.10.088 (PMC3573233; doi:10.1016/j.neuroimage.2012.10.088)
Supplement: Supplementary file 1 — Supplementary material. [file mmc1.pdf]

Supplementary Material:

Basal ganglia-cortical interactions in Parkinsonian patients

André C. Marreiros[1], Hayriye Cagnan[1], Rosalyn J. Moran[2], Karl J. Friston[2], Peter Brown[1]

[1]Department of Clinical Neurology, University of Oxford, UK

[2]The Wellcome Trust Centre for Neuroimaging, University College London, UK

Content list:

Supplemental Experimental Procedures

Supplemental Results: Model validity and reproducibility

Supplemental Table 1

Supplemental Table 2

Supplemental Figure 1

Supplemental Figure 2

Supplemental Figure 3

Supplemental Figure 4

Supplemental Figure 5

Supplemental Figure 6

Supplemental Figure 7

Supplemental Experimental Procedures

Dynamic Causal Modelling for Steady State Responses

DCM is a general framework for inferring processes and mechanisms at the neuronal level from measurements of brain activity with different techniques (Marreiros *et al.,* 2010), including fMRI (Friston *et al.,* 2003), EEG/MEG (David *et al.*, 2006; Kiebel *et al.*, 2008) and frequency spectra based on local field potentials (Moran *et al.,* 2007). In the case of LFP and EEG data, this generative model contains details about the structure and synaptic properties within each source; as well as the synaptic input that one source receives from another. DCM for steady-state responses (as employed in our analysis) rests on a biologically plausible neural mass model that can generate frequency output across the range of electrophysiological interest. The architecture shown in Supplemental Figure 1 outlines the cell types and differential equations within this neural-mass model of cortico-basal ganglia-thalamocortical circuit, presented in detail elsewhere (Moran *et al.*, 2011). This model uses intrinsic coupling parameters, γ_i, between the three subpopulations used to model cortical activity. These are connected using established connectivity rules (Felleman *et al.,* 1991). A single population of neurons, either glutamatergic (excitatory) or GABAergic (inhibitory) was used for the BG nuclei. The three subpopulations used to model cortical activity included an inhibitory subpopulation in the supragranular layer and excitatory pyramidal (output) subpopulation in an infra-granular layer. Both these subpopulations are connected to an excitatory spiny (input) subpopulation in the granular layer. Subcortical nuclei are modelled as single subpopulations. The equations are equivalent to a post-synaptic convolution of presynaptic input (firing rates) to produce depolarisation, mediated by a postsynaptic (alpha) kernel, where the presynaptic inputs are a sigmoid (voltage-firing) function of depolarisation in other neural masses (Moran *et al.,* 2008). The connection parameters act as multiplicative gain factors on the influence of firing rate from efferent ensembles. The neural mass model is used to determine how random neuronal fluctuations are filtered to produce observed cross-spectra. This entails modelling the mapping between the spectral density of neuronal fluctuations and

the resulting responses. By inverting the model, we optimise model parameters to explain empirical cross-spectra generated through kernels or transfer functions that couple the spectra of neuronal fluctuations to observed cross-spectra. We assume the observed cross-spectra are a mixture of predictions and Gaussian error,

$$y_{ij}(w) = g_{ij}^{v}(\omega,\theta) + g_{ij}^{w}(\omega,\theta) + \varepsilon_{ij}(\omega)$$

$$\varepsilon_{ij}(\omega) \sim N(0,\Sigma(\xi))$$

The spectral prediction $g_{ij}^{v}(\omega) + g_{ij}^{w}(\omega)$ comprises two parts: The first $g_{ij}^{v}(\omega)$ corresponds to cross-spectra due to neuronal activity, while the second, $g_{ij}^{w}(\omega)$ corresponds to cross spectra induced by channel noise $w(t) \in \Re$. The error $\varepsilon(\omega)$ has a covariance matrix $\Sigma(\xi) = \exp(\xi)V(\omega)$ where ξ are unknown covariance parameters and $V(t) \in \Re^{23x23}$ encodes correlations over nearby frequencies. Furthermore, we modelled common BG noise components due to volume conduction in the BG. The ensuing predictions are given by standard linear systems theory:

$$g_{ij}^{v}(\omega,\theta) = \sum_{k} \left|\Gamma_i^k(\omega,\theta).\Gamma_j^k(\omega,\theta)\right| g_k^u(\omega,\theta)$$

$$\Gamma_i^k(\omega,\theta) = \int \kappa_i^k(\tau,\theta)e^{-j\omega\iota}d\tau$$

$$g_k^u(\omega,\theta) = \alpha_k^u + \frac{\beta_k^u}{\omega}$$

$$g_{ij}^{w}(\omega,\theta) = \alpha_k^w + \frac{\beta_k^w}{\omega}$$

Here $g_{ij}^{v}(\omega,\theta)$ is the sum of cross-spectral densities induced by the inputs or neuronal fluctuations (i.e. the baseline neuronal activity which comprises a mixture of white and pink spectral components) $u_k(t)$ driving neuronal dynamics. These cross-spectra are simply the (complex) transfer functions $\Gamma_i^k(\omega,\theta) \in C^{23}$ mapping from the *k-th* neuronal fluctuations (*k*: 1,..5) to each channel times the

spectral density of each input: $g_k^u(\omega,\theta)$. We parameterised the spectra of the neuronal fluctuations and the channel noise as a mixture of white and pink components (Stevens *et al.*, 1972; Freeman *et al.*, 2003). The transfer functions are the Fourier transforms of the corresponding first-order kernels, $\kappa_i^k(\tau,\theta)$ that mediate the effect of the *k-th* innervation (zero mean fluctuations, $u_k(t)$, which we assume perturb the system linearly around its fixed point) on the observed data. These kernels can be regarded as impulse response functions of the *i-th* channel to the *k-th* input; i.e., the change in output with respect to a change in input at time τ in the past. First order kernels are ubiquitous representations of dynamical systems, where the response (of linear systems) can be determined by convolving the input with the system's impulse response or kernel. The kernel for each channel obtains analytically from the Jacobian $\partial f/\partial x$ of the flow or motion $\dot{x} = f\left(x(t), u(t), \theta\right)$ of hidden neuronal states specified by a neural mass model (see below). This flow describes how the hidden neuronal states are perturbed by the inputs or fluctuations. For channel *i*, and input *k* the kernel is

$$
\begin{aligned}
k_i^k(\tau,\theta) &= \frac{\partial z_i(t)}{\partial u_k(t-\tau)} \\
&= \frac{\partial z_i(t)}{\partial x(t)} \cdot \frac{\partial x(t)}{\partial x(t-\tau)} \cdot \frac{\partial x(t)}{\partial \dot{x}(t-\tau)} \cdot \frac{\partial \dot{x}(t-\tau)}{\partial u_k(t-\tau)} \\
&= \frac{\partial h_i(t)}{\partial x(t)} \cdot \exp\left(\tau \frac{\partial f}{\partial x}\right) \cdot \frac{\partial f}{\partial x}^{-1} \cdot \frac{\partial f(t)}{\partial u_k(t)}
\end{aligned}
$$

This means the kernels are analytic functions of the equations of motion of the hidden states, the mapping between hidden states and inputs. In addition, we have to model the mapping between hidden states and observed channel data in the time domain, $z(t) = h(x(t),\theta) + w(t)$, which forms the basis of the spectral prediction. The observation function $h(x(t),\theta)$ used here is a simple weighted mixture of depolarisations at pyramidal (60%), stellate (20%) and inhibitory interneurons (20%) contributing to the cortical EEG channel and the depolarization of the individual cell populations in each BG recording. We consider a linear mapping from *s* sources to *c* channels. In EEG this mapping is a lead-field or gain-matrix function, $L(\theta) \in \Re^{cxs}$, of unknown spatial

parameters, θ, such as source location and orientation. For invasive LFP recordings that are obtained directly from the neuronal sources, this mapping is a leading diagonal gain-matrix, $L = diag(\theta_1,...\theta_s)$ where the parameters model electrode-specific gains

In our DCM, each region receives an identical mixture of white and pink noise, inferred from the data. Also, each recording is assumed to contain random (white) and autoregressive (pink) channel noise. This channel noise is modelled as having a constant, channel independent component and either a BG (DBS electrode) or cortical (EEG electrode) component. These approximations served to account for channel and instrumentation noise and volume conduction. There were some differences between the priors in the patient and our previous rodent models (Moran *et al.*, 2011). These related to differences in recording techniques and were at the level of the parameters controlling the spectral composition of neuronal and channel noise, which separately control the mixture of white and pink noises assumed to exist in the BG and cortical channels. Both were changed to optimize the DCM fit to the human data spectrum. The covariance priors of these last parameters were also relaxed to account for differences arising from the bigger electrodes used in patients than in rodents, thereby allowing more flexibility into the DCM estimation process.

Bayesian Model Inversion and comparison

For a given DCM, say model *m*, parameter estimation corresponds to approximating the moments of the posterior distribution given by Bayes rule

$$p(\theta \mid y, m) = \left(p(y \mid \theta, m)p(\theta \mid m)\right)/p(y \mid m)$$

This scheme is identical to that employed by DCM for fMRI and evoked responses (Fahrmeir *et al.*, 2001; Friston *et al.*, 2007), and has been previously described in detail (Moran *et al.*, 2007). The posterior moments (conditional mean η and covariance Ω) are updated iteratively using a variational

scheme under a fixed-form Laplace (*i.e.*, Gaussian) approximation to the conditional density $q(\theta) = N(\eta,\Omega)$. This can be regarded as an Expectation-Maximization (EM) algorithm that employs a local linear approximation of the predicted responses about the current conditional expectation. The **E**-step conforms to a Fisher-scoring scheme (Moran *et al.*, 2008) that performs a descent on a variational free-energy $F(q,\lambda,m)$, with respect to the conditional moments. In the **M**-Step, the error variances λ are updated in exactly the same way to provide their maximum likelihood. The estimation procedure employed in DCM has been previously described (Friston *et al.*, 2003) and can be summarized as follows:

$$\textbf{E}\text{-step: } q \leftarrow \min_q F(q,\lambda,m)$$

$$\textbf{M}\text{-step: } \lambda \leftarrow \min_\lambda F(q,\lambda,m)$$

$$\begin{aligned} F(q,\lambda,m) &= \left\langle \ln q(\vartheta) - \ln p(y|\vartheta,\lambda) - \ln p(\vartheta|m) \right\rangle_q \\ &= KL(q|p(\vartheta|y,\lambda)) - \ln(p(y|\lambda,m)) \end{aligned}$$

The free-energy is simply a function of the log-likelihood, the log-prior and the approximation to the conditional density we seek. The free-energy is the Kullback–Leibler divergence between the real and approximate posterior or conditional density, minus the log-likelihood. This means that when the free-energy is minimised, the discrepancy between the true and approximate conditional density is suppressed (because the divergence is non-negative). At this point the free-energy approximates the negative log-evidence $F \approx -lnp(y|\lambda,m)$, where λ are the unknown covariance component parameters; i.e., the hyperparameters (Stevens *et al.*, 1972). This is the maximum value of the objective function attained by **EM**. The most likely model is the one with the largest log-evidence. Model comparison rests on the likelihood ratio of the evidence for two models. This ratio is the Bayes factor B_{ij}. For models *i* and *j*

$$lnB_{ij} = \ln p(y|m = i) - \ln p(y|m = j)$$

Conventionally, strong evidence in favour of one model requires the difference in log-evidence to be about three or more (Penny *et al.*, 2004). Under the assumption that all models are equally likely *a priori*, the marginal densities $p(y|m)$ can be converted into the probability of the model given the data $p(m|y)$ (by normalising so that they sum to one over models). It is this probability that we use to quantify model comparisons.

Supplemental Results: Model validity and reproducibility

In this study we used an exceptional archival data set from a group of patients with Parkinson's Disease who underwent simultaneous implantation of deep brain stimulation (DBS) electrodes into the GPi and the STN and recording of electroencephalographic activity (EEG). The patients were enrolled in a trial of combined pallidal and subthalamic DBS (Peppe et al., 2004). The trial has since closed with essentially negative clinical results, and we have not had the opportunity to simultaneously record from these particular sites again. Thus we cannot directly test our model against another clinical data set. However, the same model architecture previously fitted data from parkinsonian rodents (Moran *et al.*, 2011). In addition, as a test of the validity of our model we demonstrated that lesioning of the STN, and of pathways to and from it, resulted in suppression of beta activity in the whole circuit, in line with the clinical effects of such interventions (see Results and Discussion).

To further address the issue of reproducibility we took the two subjects with the longest data recordings, halved their data and fitted our model to each data half. The two fits from one of these subjects are shown in Supplemental Figure 3A and B. Separate auto-spectral and cross-spectral densities from cortex, STN and GPi over 13-35 Hz from ON and OFF medication are shown together as full lines in each panel. The 95% confidence intervals of the corresponding DCM predictions are shown as shaded areas. The main diagonal displays the auto-spectral densities at each site and the off-diagonal elements shows the cross spectra. Empirical spectra generally fall inside the shaded areas

and indicate an overall good model fit with respect to both data sets. Our original group analysis highlighted increases in the maximum a posteriori (MAP) connection strengths from GPe to STN, cortex to STN and STN to GPi from ON to OFF levodopa. Within this subject all the three of these connections were strengthened when the model was fitted to the data half in A and the data half in B.

The corresponding findings are illustrated for the second patient in Supplemental Figure 4A and B. The model fit to the first data half was good, and maximum a posteriori (MAP) connection strengths were significantly strengthened for two of the key connections (cortex to STN and STN to GPi) in the OFF levodopa state. The model fit to the second data half was less good although spectral peak frequencies were still picked up. The confidence limits of the MAP estimates were correspondingly wide, so that none of the key connections were identified as significantly changed. This variability between samples from the same subject might reflect slightly different levels of arousal at rest or the limitations of fitting with half the former data sample.

References

David, O., Harrison, L. & Friston, K.J. (2005). Modelling event-related responses in the brain. NeuroImage *25*, 756-770.

David, O., Kiebel, S.J., Harrison, L.M., Mattout, J., Kilner, J.M., Friston, K.J. (2006). Dynamic causal modeling of evoked responses in EEG and MEG. NeuroImage, *30*:1255-72.

Fahrmeir, L. & Tutz, G. (2001). Multivariate Statistical Modelling Based on Generalized Linear Models. Journal of the American Statistical Association *91*, 425 p. (Springer).

Felleman, D. J., & Van Essen, D. C. (1991). Distributed hierarchical processing in the primate cerebral cortex. Cerebral Cortex, *1(1)*, 1-47.

Freeman, W.J., Holmes, M.D., Burke, B.C. & Vanhatalo, S. (2003). Spatial spectra of scalp EEG and EMG from awake humans. Clinical Neurophysiology *114*, 1053-1068.

Friston, K.J., Harrison, L., and Penny, W. (2003). Dynamic causal modelling. Neuroimage, *19*, 1273-1302.

Friston, K., Mattout, J., Trujillo-Barreto, N., Ashburner, J. & Penny, W. (2007). *Variational free energy and the Laplace approximation*. NeuroImage *34*, 220-234.

Jansen BH, Rit VG (1995) Electroencephalogram and visual evoked potential generation in a mathematical model of coupled cortical columns. Biol Cybern *73*:357–366.

Kiebel, S. J., Garrido, M. I., Moran, R. J., & Friston, K. J. (2008). Dynamic causal modelling for EEG and MEG. Cognitive neurodynamics, *2(2)*, 121-136.

Marreiros, A.C., Friston, K.J. & Stephan, K.E. (2010). Dynamic causal modeling. Scholarpedia *5*,9568.

Moran, R.J., Kiebel, S.J., Stephan, K.E., Reilly, R.B., Daunizeau, J., Friston, K.J. (2007). A neural mass model of spectral responses in electrophysiology. Neuroimage *37*, 706-20.

Moran, R J, Stephan, K. E., Kiebel, S. J., Rombach, N., O'Connor, W. T., Murphy, K. J., Reilly, R. B., et al. (2008). Bayesian estimation of synaptic physiology from the spectral responses of neural masses. NeuroImage, *42(1)*, 272-284.

Moran, R.J., Mallet, N., Litvak, V., Dolan, R.J., Magill, P.J., Friston, K.J., Brown, P. (2011). Alterations in brain connectivity underlying Beta oscillations in Parkinsonism. PLoS Computational Biology *7*, e1002124.

Peppe A., Pierantozzi M., Bassi A., Altibrandi M.G., Brusa L., Stefani A., et al. (2004) Stimulation of the subthalamic nucleus compared with the globus pallidus internus in patients with Parkinson disease. Journal of Neurosurgery, *101*: 195-200.

Penny, W.D., Stephan, K.E., Mechelli, A. & Friston, K.J. (2004). Comparing dynamic causal models. NeuroImage *22*, 1157-1172.

Stevens, C.F. (1972). Inferences about membrane properties from electrical noise measurements. Biophysical journal *12*, 1028–1047.

Supplemental Table 1. **Priors for model parameters including the observation model and neuronal sources.**

Parameter<br>$\vartheta_i = \pi_i \exp(\Theta_i)$<br>$\Theta_i = N(0, \sigma_i)$	Interpretation	Prior<br>Mean:<br>π_i	 Variance:<br>σ_i
**Neuronal Sources**			
	Sigmoid		
ρ_1	*Shape parameter*	*0*	*1/2*
ρ_2	*Position parameter*	*0*	*1/2*
$H_{e/i}$	***post-synaptic potentials (mV)***		
	Maximum Excitatory	*8*	*1/2*
	Maximum Inhibitory	*32*	*1/2*
$\kappa_{e/i}$	***Rate Constants (ms^{-1})***		
	Excitatory	*4*	*1/2*
	Inhibitory	*16*	*1/2*

$\gamma_{1,2,3,4,5}$	***Intrinsic Cortical Connections***		
	1. Pyramidal cells to stellate cells	*128*	*1*
	2. Stellate cells to Pyramidal cells	*128*	*1*
	3. Pyramidal cells to Inhibitory interneurons	*64*	*1*
	4. Inhibitory interneurons to Pyramidal cells	*64*	*1*
	5. Reciprocal Inhibitory connections	*4*	*1*
	Extrinsic Connections		
$\lambda_{A,B}$	*Excitatory*	*32*	*1/2*
$\lambda_{A,B}$	*Inhibitory*	*16*	*1/2*
λ_C	*Input connections*	*0*	*1/32*
	Delays (ms)		
D_{ext}	*Extrinsic*	*4*	*1/8*
d_{int}	*Intrinsic (cortical layers)*	*2*	*1/32*
	Exogenous Input		
α_u	*White*	*-4*	*7*
β_u	*Pink*	*-4*	*7*
**Observation Model**			
	Noise at cortical channel		

α_{ctx}	*White*	*-1*	*4*
β_{ctx}	*Pink*	*-2*	*4*
	Noise at BG channel		
α_{BG}	*White*	*0*	*4*
β_{BG}	*Pink*	*0*	*4*
	Noise common to all BG channels		
α_{comm}	*White*	*-1*	*7*
β_{comm}	*Pink*	*-2*	*7*

Supplemental Table 1. Priors for model parameters including the observation model and neuronal sources. Parameters are optimised by multiplying their prior expectation with an unknown log-scale parameter that is exponentiated to ensure positivity. Hence, a log-scale parameter of zero corresponds to a scale-parameter of one, which renders the parameter value equal to its prior expectation. By imposing Gaussian priors on the log-scale parameters we place log-normal priors on the parameters *per se.* In practice, it assumes a Gaussian density on a scale parameter, $\Theta_i = N(0,\sigma_i^2)$, where $\vartheta_i = \pi_i \exp(\Theta_i)$, and π_i is the prior expectation and σ_i^2 is its log-normal dispersion (David et al., 2005).

Supplemental Table 2. Comparison of findings in PD patients with those in 6OHDA midbrain lesion rodents

	**PD Patients**	**6OHDA midbrain lesioned**

		**rodents**
**Change in Connection strength from ON to OFF in PD or from healthy to lesioned in rodents**	*Ctx-STN strengthened*<br><br>STN-GPi strengthened<br><br>GPe- STN strengthened	*Ctx-STN strengthened*<br><br>STN-GPe weakened
**Change in Connection contribution from ON to OFF in PD or from healthy to lesioned in rodents**	Ctx-STN increased<br><br>*GPe-STN increased*<br><br>STN-GPe increased<br><br>STN-GPi increased	Striatum-GPe increased<br><br>*GPe-STN increased*

Supplemental Table 2. Comparison of findings in PD patients with those in 6OHDA midbrain lesion rodents

Schematic summary of significant changes in effective connectivity and contribution. Both models showed strengthening of the hyperdirect pathway in the Parkinsonian state and increased beta promoting potency in the GPe to STN pathway.

Supplemental Figure 1. Schematic of the neural-mass model of cortico-basal ganglia thalamocortical circuit

Schematic of the DCM used to model electrophysiological responses. This schematic shows the state equations describing the dynamics of sources or regions. Each source is modelled with up to three subpopulations (pyramidal, spiny stellate and inhibitory interneurons) (Jansen *et al.*, 1995).

Supplemental Figure 2. Model evidence for DCMs

Here, we show the model evidence (as approximated with the negative free energy, F) for all DCMs in our study. The accuracy or model fit for three data segments was poor and these DCMs were not included in subsequent analyses. However, all patients contributed at least one data segment to subsequent analyses.

Supplemental Figure 3. Reproducibility in case 1.

Data from case 1 have been halved in (A) and (B). Separate auto-spectral and cross-spectral densities from cortex, STN and GPi over 13-35 Hz from ON and OFF medication are shown together as dotted lines in each panel. The 95% confidence intervals of the corresponding DCM predictions are shown as shaded areas. The main diagonal displays the auto-spectral densities at each site and the off-diagonal elements shows the cross spectra.

Supplemental Figure 4. Reproducibility in case 2.

Data from case 2 have been halved in (A) and (B). Separate auto-spectral and cross-spectral densities from cortex, STN and GPi over 13-35 Hz from ON and OFF medication are shown together as dotted lines in each panel. The 95% confidence intervals of the corresponding DCM predictions are shown as shaded areas. The main diagonal displays the auto-spectral densities at each site and the off-diagonal elements shows the cross spectra.

Supplemental Figure 5. Posterior correlations and parameter identifiability

Here, we show the posterior correlation matrices derived from the posterior covariance of the averaged ON and OFF state DCMs. High correlations in posterior estimates indicate redundancy in the network, where two parameters may contribute equivalently to the observed response. The colour bar is applicable to all images. *Left.* For our parameters of interest; the modulation of the connectivity

parameters, the average (absolute) correlation of parameters was only 0.06 ± 0.01 (Mean ± SEM, n = 64). See Supplemental Table 1 for explanation of symbols.

Supplemental Figure 6. Priors and posteriors

Under Gaussian assumptions, the prior distribution $p(\theta)$ is defined by its mean and covariance Σ. Here we plot the group averaged priors and their 95% confidence limits (in blue) and the corresponding group averaged posteriors and their 95% confidence limits (in red) obtained after inverting the model for all biophysical and connectivity parameters. The neuronal priors scaling parameters are set to unity, so that experimental conditions (ON and OFF levodopa) are modelled by coupling gains, thus acting as trial-specific scaling multiplicative factors. Only one parameter, the 'GPi intrinsic reciprocal connection' posterior, is significantly different to its prior value and the absolute mean difference between posteriors and priors is 0.40 ± 0.08 (Mean ± SEM, n = 73). See Supplemental Table 1 for explanation of symbols.

Supplemental Figure 7. Lesion analysis

(**A**) Effects of separately resetting CTX-STN, GPe-STN, STN-GPe and STN-GPi to ON state values, whilst leaving all other connections with their OFF drug strengths; and of separately lesioning CTX-STN and STN-GPi. Spectra of beta responses in the Parkinsonian network before and after resetting or lesioning are shown above in red and blue respectively. (**B**) Below are the corresponding Wilcoxon signed-rank tests of the effects on resetting or lesioning. Beta activity was profoundly and significantly suppressed when one or other of the recurrent connections between STN and GPe were reset. The hyperdirect CTX-STN and the STN-GPi connections were also important for sustaining beta oscillations, although these connections had to be lesioned in order to achieve significant attenuation of beta activity in the system. Horizontal red lines denote $p < 0.01$ (Wilcoxon signed-rank tests across frequencies).

SuppFig. 1

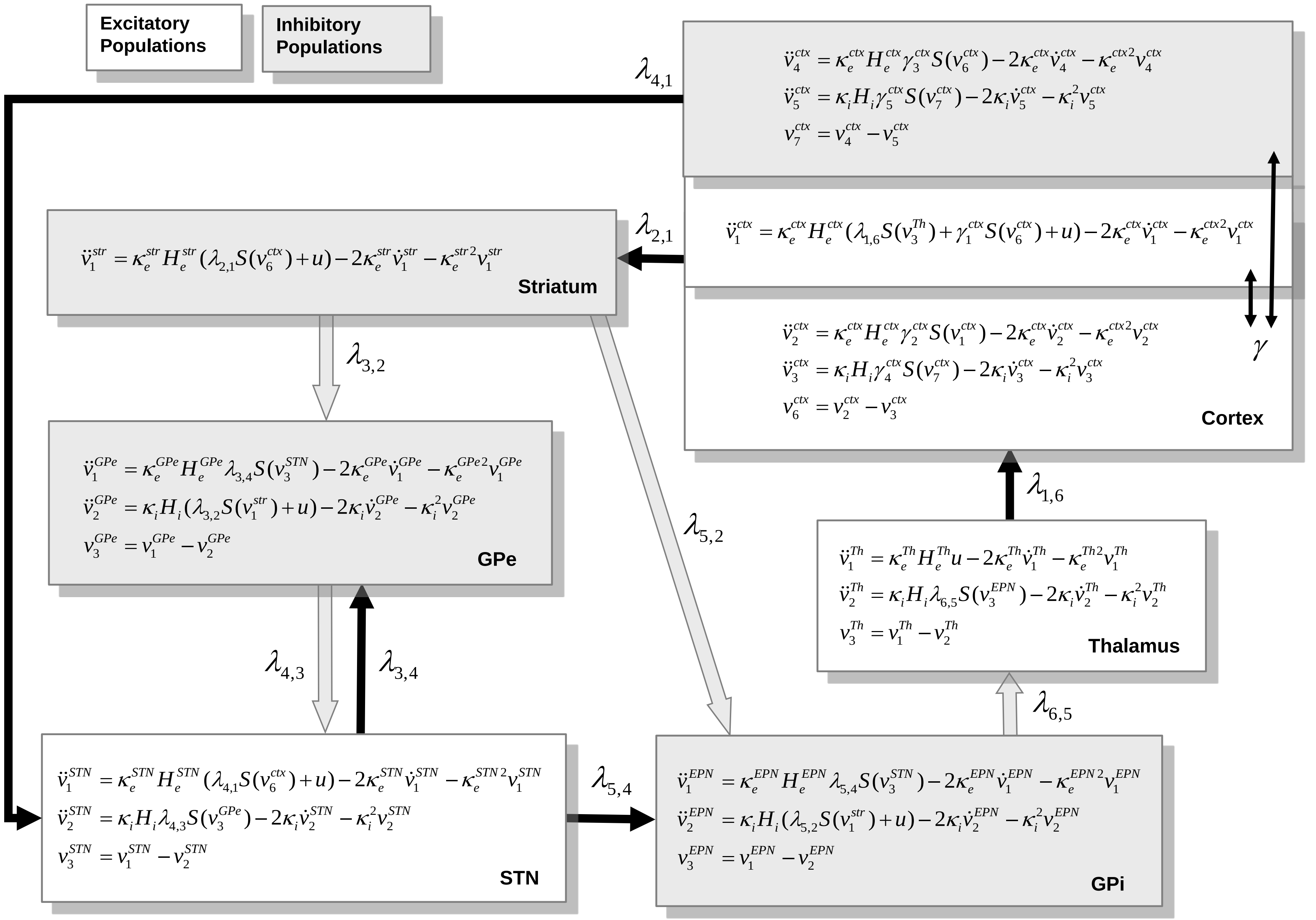

Excitatory Populations
Inhibitory Populations
$\lambda_{4,1}$
$\ddot{v}_4^{ctx} = \kappa_e^{ctx} H_e^{ctx} \gamma_3^{ctx} S(v_6^{ctx}) - 2\kappa_e^{ctx} \dot{v}_4^{ctx} - \kappa_e^{ctx2} v_4^{ctx}$
$\ddot{v}_5^{ctx} = \kappa_i H_i \gamma_5^{ctx} S(v_7^{ctx}) - 2\kappa_i \dot{v}_5^{ctx} - \kappa_i^2 v_5^{ctx}$
$v_7^{ctx} = v_4^{ctx} - v_5^{ctx}$
$\lambda_{2,1}$
$\ddot{v}_1^{ctx} = \kappa_e^{ctx} H_e^{ctx} (\lambda_{1,6} S(v_3^{Th}) + \gamma_1^{ctx} S(v_6^{ctx}) + u) - 2\kappa_e^{ctx} \dot{v}_1^{ctx} - \kappa_e^{ctx2} v_1^{ctx}$
$\ddot{v}_1^{str} = \kappa_e^{str} H_e^{str} (\lambda_{2,1} S(v_6^{ctx}) + u) - 2\kappa_e^{str} \dot{v}_1^{str} - \kappa_e^{str2} v_1^{str}$
Striatum
$\ddot{v}_2^{ctx} = \kappa_e^{ctx} H_e^{ctx} \gamma_2^{ctx} S(v_1^{ctx}) - 2\kappa_e^{ctx} \dot{v}_2^{ctx} - \kappa_e^{ctx2} v_2^{ctx}$
$\ddot{v}_3^{ctx} = \kappa_i H_i \gamma_4^{ctx} S(v_7^{ctx}) - 2\kappa_i \dot{v}_3^{ctx} - \kappa_i^2 v_3^{ctx}$
$v_6^{ctx} = v_2^{ctx} - v_3^{ctx}$
γ
Cortex
$\lambda_{3,2}$
$\ddot{v}_1^{GPe} = \kappa_e^{GPe} H_e^{GPe} \lambda_{3,4} S(v_3^{STN}) - 2\kappa_e^{GPe} \dot{v}_1^{GPe} - \kappa_e^{GPe2} v_1^{GPe}$
$\ddot{v}_2^{GPe} = \kappa_i H_i (\lambda_{3,2} S(v_1^{str}) + u) - 2\kappa_i \dot{v}_2^{GPe} - \kappa_i^2 v_2^{GPe}$
$v_3^{GPe} = v_1^{GPe} - v_2^{GPe}$
GPe
$\lambda_{1,6}$
$\lambda_{5,2}$
$\ddot{v}_1^{Th} = \kappa_e^{Th} H_e^{Th} u - 2\kappa_e^{Th} \dot{v}_1^{Th} - \kappa_e^{Th2} v_1^{Th}$
$\ddot{v}_2^{Th} = \kappa_i H_i \lambda_{6,5} S(v_3^{EPN}) - 2\kappa_i \dot{v}_2^{Th} - \kappa_i^2 v_2^{Th}$
$v_3^{Th} = v_1^{Th} - v_2^{Th}$
Thalamus
$\lambda_{4,3}$
$\lambda_{3,4}$
$\lambda_{6,5}$
$\ddot{v}_1^{STN} = \kappa_e^{STN} H_e^{STN} (\lambda_{4,1} S(v_6^{ctx}) + u) - 2\kappa_e^{STN} \dot{v}_1^{STN} - \kappa_e^{STN2} v_1^{STN}$
$\ddot{v}_2^{STN} = \kappa_i H_i \lambda_{4,3} S(v_3^{GPe}) - 2\kappa_i \dot{v}_2^{STN} - \kappa_i^2 v_2^{STN}$
$v_3^{STN} = v_1^{STN} - v_2^{STN}$
STN
$\lambda_{5,4}$
$\ddot{v}_1^{EPN} = \kappa_e^{EPN} H_e^{EPN} \lambda_{5,4} S(v_3^{STN}) - 2\kappa_e^{EPN} \dot{v}_1^{EPN} - \kappa_e^{EPN2} v_1^{EPN}$
$\ddot{v}_2^{EPN} = \kappa_i H_i (\lambda_{5,2} S(v_1^{str}) + u) - 2\kappa_i \dot{v}_2^{EPN} - \kappa_i^2 v_2^{EPN}$
$v_3^{EPN} = v_1^{EPN} - v_2^{EPN}$
GPi

SuppFig. 2

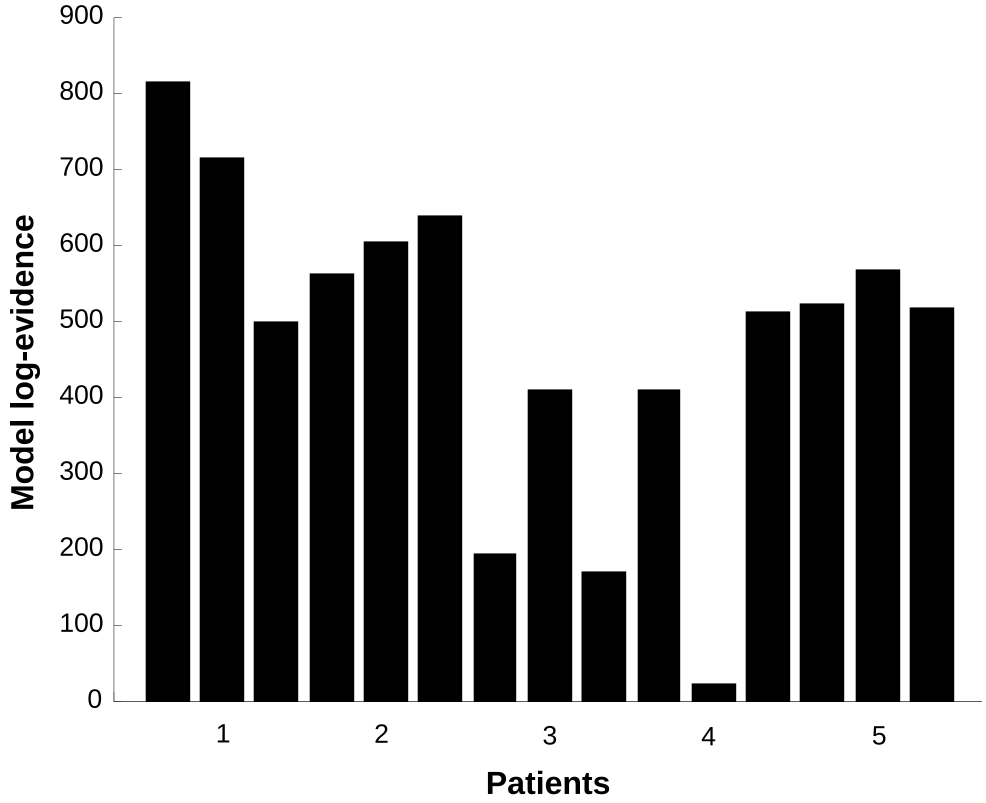

Model log-evidence
900
800
700
600
500
400
300
200
100
0
1
2
3
4
5
Patients

SuppFig. 3 **A**

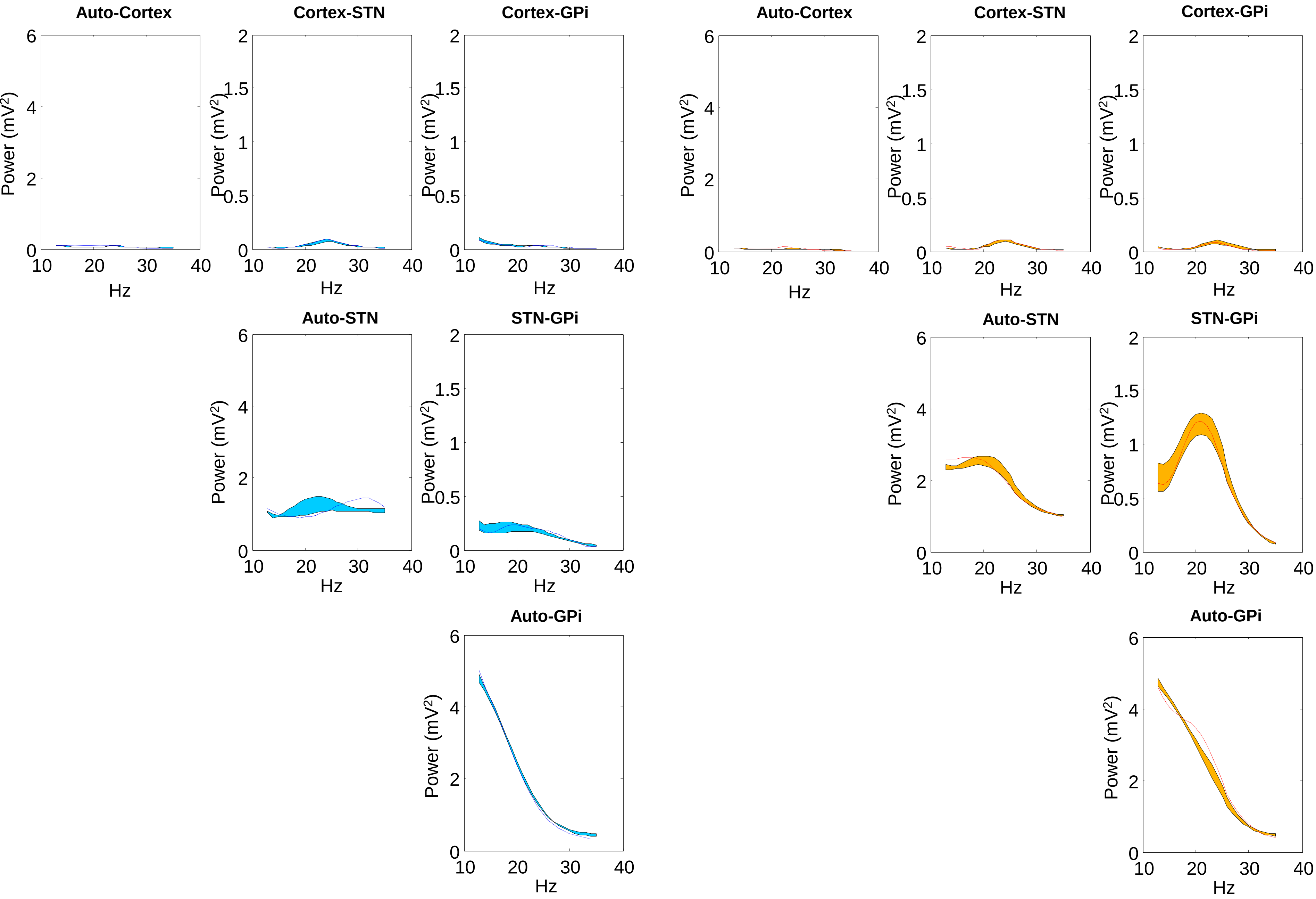

Auto-Cortex
Cortex-STN
Cortex-GPi
Auto-Cortex
Cortex-STN
Cortex-GPi
Auto-STN
STN-GPi
Auto-STN
STN-GPi
Auto-GPi
Auto-GPi
Power (mV²)
Hz

SuppFig. 3 **B**

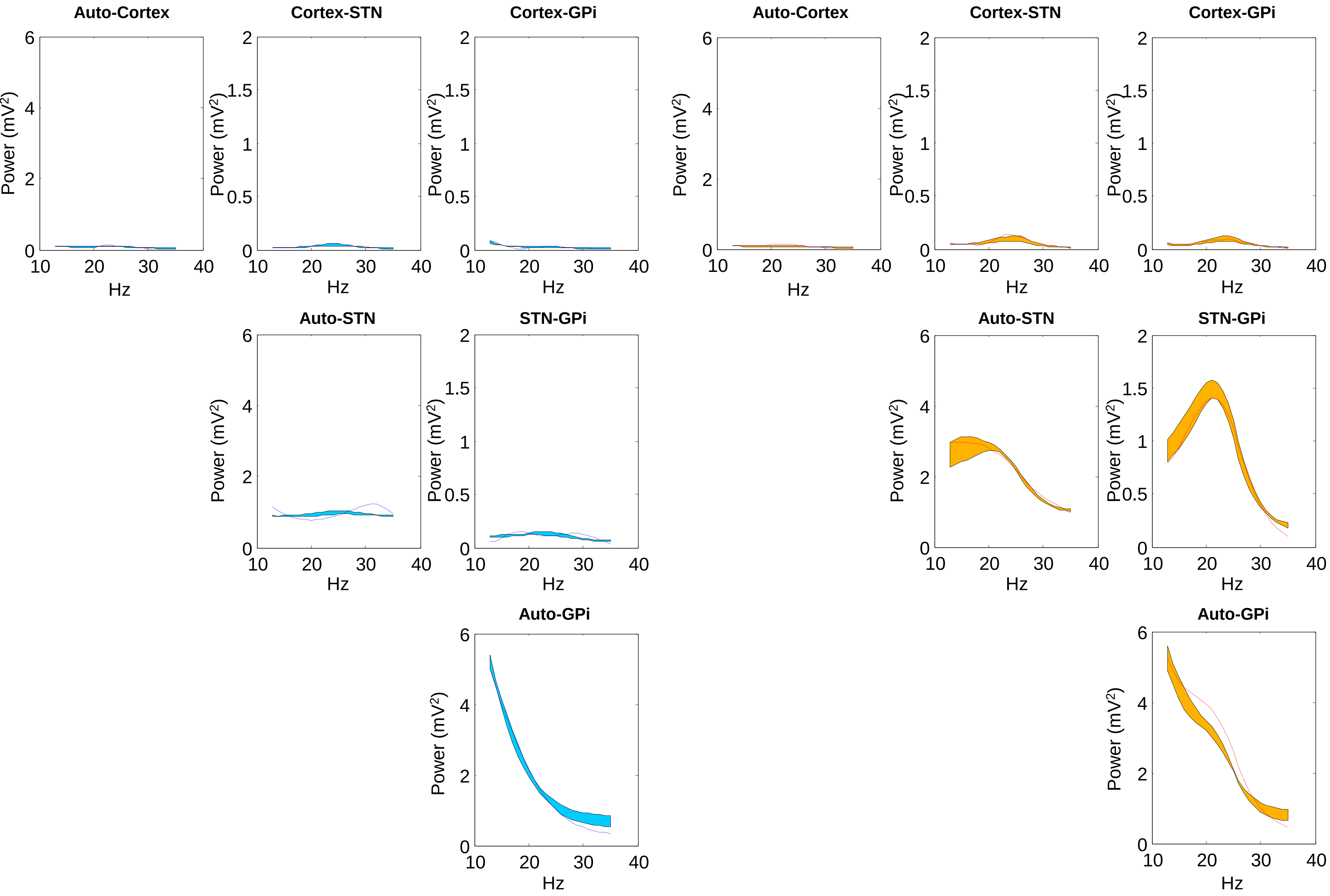

Auto-Cortex
Cortex-STN
Cortex-GPi
Auto-Cortex
Cortex-STN
Cortex-GPi
Auto-STN
STN-GPi
Auto-STN
STN-GPi
Auto-GPi
Auto-GPi
Power (mV²)
Hz

SuppFig. 4 **A**

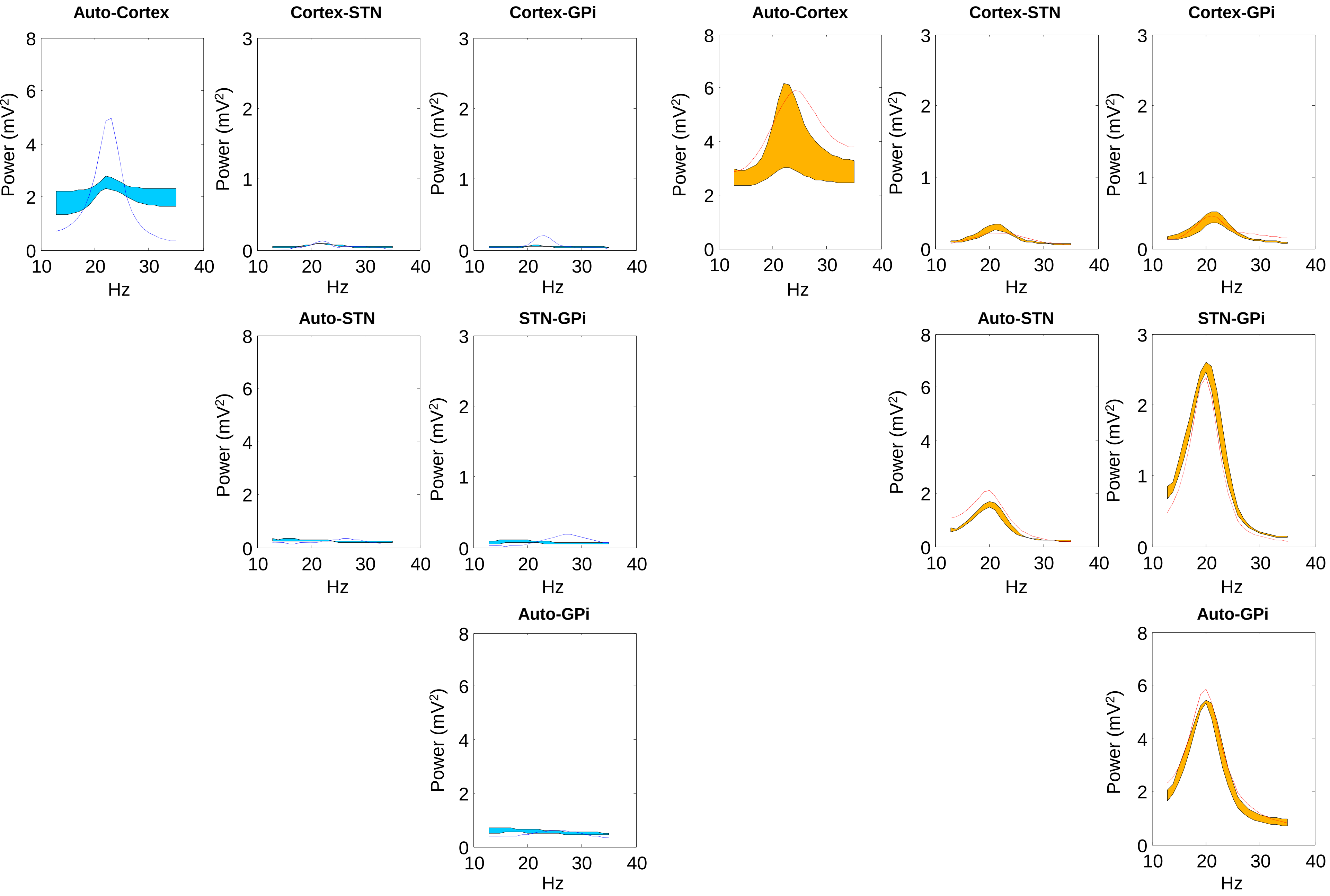

Auto-Cortex
Cortex-STN
Cortex-GPi
Auto-Cortex
Cortex-STN
Cortex-GPi
Auto-STN
STN-GPi
Auto-STN
STN-GPi
Auto-GPi
Auto-GPi
Power (mV²)
Hz

SuppFig. 4 **B**

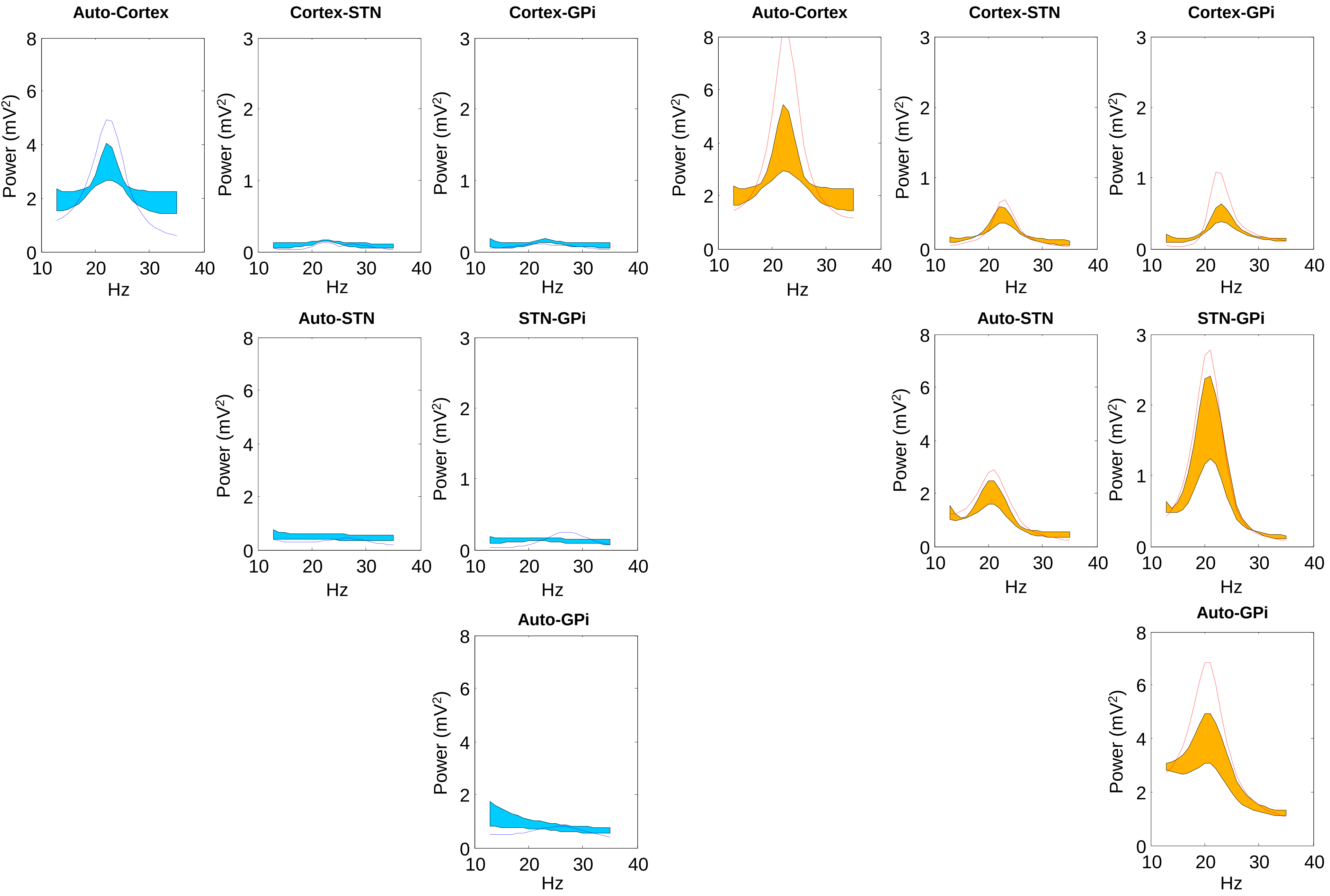

Auto-Cortex
Cortex-STN
Cortex-GPi
Auto-Cortex
Cortex-STN
Cortex-GPi
Auto-STN
STN-GPi
Auto-STN
STN-GPi
Auto-GPi
Auto-GPi
Power (mV²)
Hz

SuppFig. 5

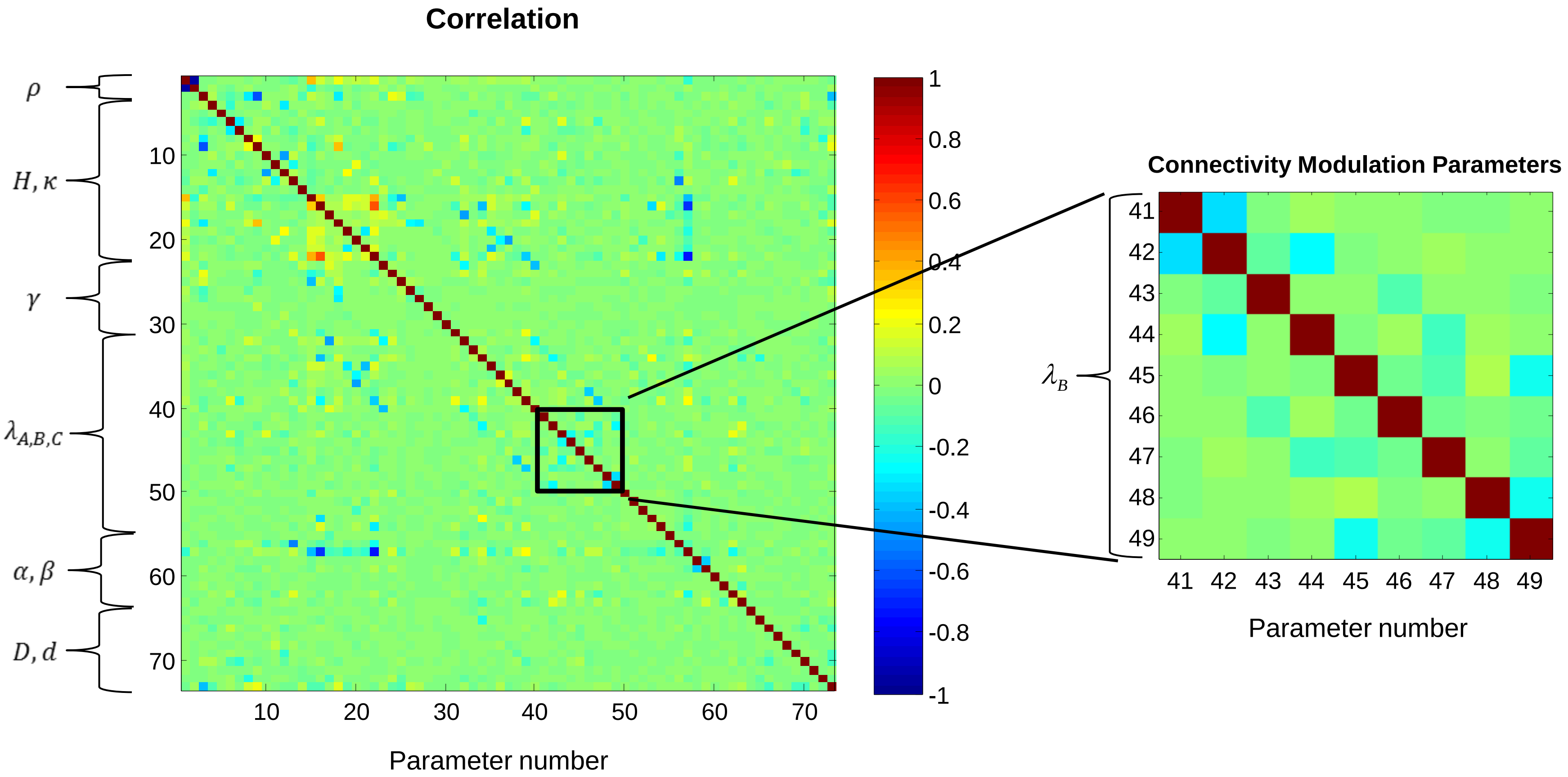
Correlation
ρ
H, κ
γ
λ_{A,B,C}
α, β
D, d
10
20
30
40
50
60
70
Parameter number
1
0.8
0.6
0.4
0.2
0
-0.2
-0.4
-0.6
-0.8
-1
Connectivity Modulation Parameters
λ_B
41
42
43
44
45
46
47
48
49
Parameter number

SuppFig. 6

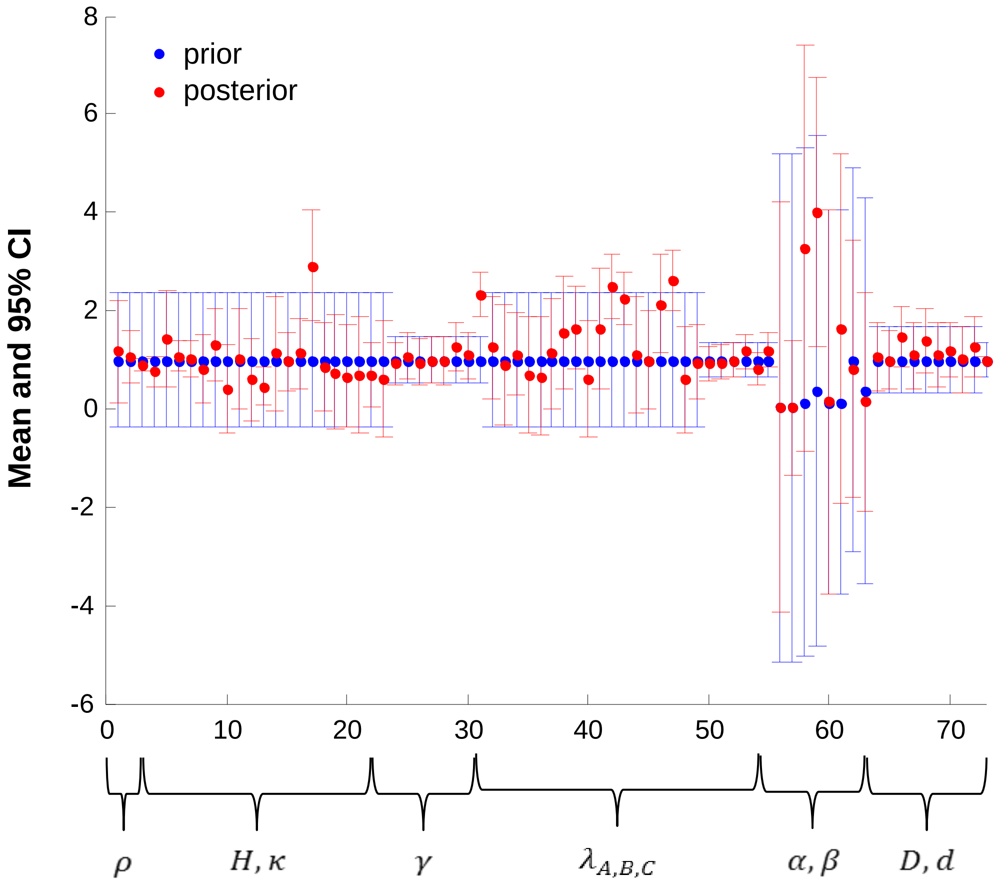

Mean and 95% CI
8
6
4
2
0
-2
-4
-6
0
10
20
30
40
50
60
70
prior
posterior
ρ
H, κ
γ
$\lambda_{A,B,C}$
α, β
D, d

SuppFig. 7

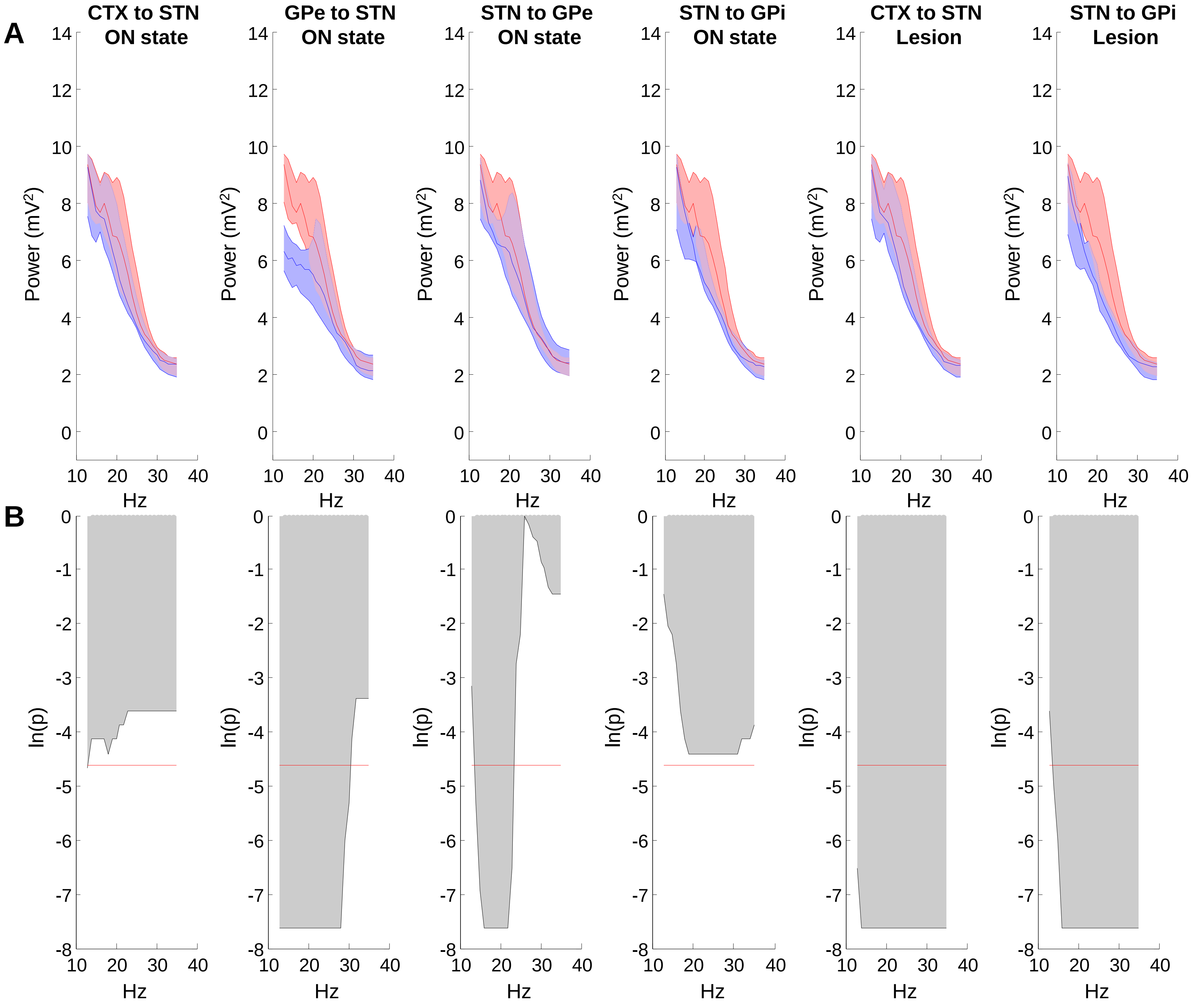
A
CTX to STN ON state
GPe to STN ON state
STN to GPe ON state
STN to GPi ON state
CTX to STN Lesion
STN to GPi Lesion
Power (mV^2)
Hz
B
ln(p)
Hz
